# HPF1 dynamically controls the PARP1/2 balance between initiating and elongating ADP-ribose modifications

**DOI:** 10.1038/s41467-021-27043-8

**Published:** 2021-11-18

**Authors:** Marie-France Langelier, Ramya Billur, Aleksandr Sverzhinsky, Ben E. Black, John M. Pascal

**Affiliations:** 1 Department of Biochemistry and Molecular Medicine, Université de Montréal, Montréal, QC H3C 3J7 Canada; 2 Department of Biochemistry and Biophysics, Penn Center for Genome Integrity, Perelman School of Medicine, University of Pennsylvania, Philadelphia, PA 19104-6059 USA

**Keywords:** PolyADP-ribosylation, Enzyme mechanisms, Structural biology

## Abstract

PARP1 and PARP2 produce poly(ADP-ribose) in response to DNA breaks. HPF1 regulates PARP1/2 catalytic output, most notably permitting serine modification with ADP-ribose. However, PARP1 is substantially more abundant in cells than HPF1, challenging whether HPF1 can pervasively modulate PARP1. Here, we show biochemically that HPF1 efficiently regulates PARP1/2 catalytic output at sub-stoichiometric ratios matching their relative cellular abundances. HPF1 rapidly associates/dissociates from multiple PARP1 molecules, initiating serine modification before modification initiates on glutamate/aspartate, and accelerating initiation to be more comparable to elongation reactions forming poly(ADP-ribose). This “hit and run” mechanism ensures HPF1 contributions to PARP1/2 during initiation do not persist and interfere with PAR chain elongation. We provide structural insights into HPF1/PARP1 assembled on a DNA break, and assess HPF1 impact on PARP1 retention on DNA. Our data support the prevalence of serine-ADP-ribose modification in cells and the efficiency of serine-ADP-ribose modification required for an acute DNA damage response.

## Abstract (web summary)

HPF1 controls the ADP-ribosylation activity of PARP1/2 in response to DNA breaks. Here, the authors show that HPF1 regulates the balance between ADP-ribose initiation and elongation through a dynamic interaction that accelerates the initiation rate on serine residues.

## Introduction

**Fig. 1:**
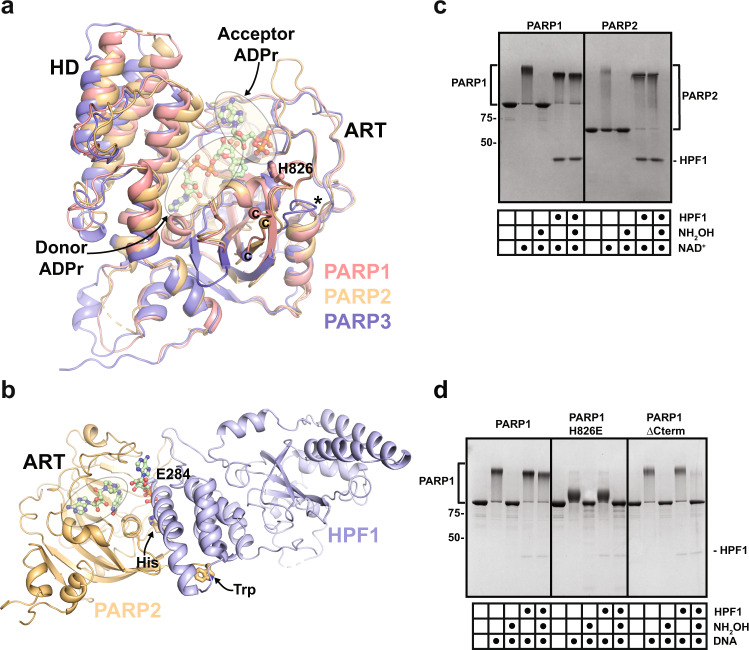
HPF1 regulation of PARP1 and PARP2. **a** The catalytic domains of PARP1 (4DQY), PARP2 (4PJV), and PARP3 (3FHB) are superimposed. The helical domain (HD) and the ADP-ribosyltransferase (ART) domain are labeled. The Donor ADPr site is defined by the PARP1 complex with BAD (6BHV), and the Acceptor ADPr site is defined by the PARP1 complex with carba-NAD^+^ (1A26). PARP1 and PARP2 share a similar structure of the loop that bears residue H826 (PARP1), whereas PARP3 has a truncated loop (*). PARP1 and PARP2 share conserved residues at their C-terminal ends that are not conserved in PARP3 (C-termini labeled “C”). **b** The ART of PARP2 (HD deleted) in complex with HPF1 (6TX3). A conserved His residue from the loop highlighted in panel B (826 in PARP1), and a Trp residue at the C-terminus (1014 in PARP1), are drawn as sticks and labeled (His and Trp). The Donor site is bound by the NAD^+^ mimic EB47 that was crystallized with the complex. HPF1 binding clashes with the Acceptor site (carba-NAD^+^ is shown bound to the acceptor site as in panel B in order to highlight the clash). HPF1 residue E284 directly contributes to the PARP active site. **c** PARP1 or PARP2 (1 μM) was incubated with dumbbell DNA containing a central nick (1 μM) with or without HPF1 (1 μM) for 10 min at room temperature (RT). 500 μM NAD^+^ was added for 5 min and reactions were quenched with 500 µM PARP inhibitor (olaparib or talazoparib). Where indicated, reactions were treated with 1 M hydroxylamine (NH_2_OH) for one hour. Reactions were resolved by SDS-PAGE and treated with Imperial Stain. **d** Same as panel **c** for PARP1 WT, mutant H826E, and mutant ΔCterm (Δ1012–1014), with 0.1 μM of HPF1. The experiment in **c** was performed three times. The experiment in **d** was performed two times. Numbers on the left side of the gels represent molecular weight marker locations (values in kDa). Source data are provided as a Source Data file.

Genome integrity in eukaryotic cells is maintained by a variety of surveillance pathways. Poly (ADP-ribose) polymerase (PARP) 1, 2, and 3 are considered first responders in the cellular response to DNA damage since they rapidly detect and signal DNA strand breaks^1^. PARP1/2/3 binding to DNA breaks strongly stimulates their catalytic activities, which consists of covalently attaching ADP-ribose to target proteins using NAD^+^ as a source of ADP-ribose. While PARP3 only adds mono-ADP-ribose (MAR) to its targets, PARP1 and PARP2 can form chains of ADP-ribose units known as poly(ADP-ribose) or PAR^2^. The catalytic domain structure of PARP1 (Fig. 1a) indicated an “acceptor” site where ADP-ribose can bind and be elongated with another ADP-ribose unit from the NAD^+^ “donor” site^3^. PARP1/2/3 modify themselves with ADP-ribose at multiple sites, a process termed auto-modification, and they also modify a variety of other proteins including histones, other DNA repair factors, and certain DNA structures^4–6^. The rapid production of PAR and MAR at and around the site of DNA damage allows recruitment of DNA repair factors harboring PAR and MAR binding domains to start the repair process. The ADP-ribose modification can also modulate the local structure of chromatin to facilitate repair, and is also proposed to seed the assembly of phase condensates that create a repair environment^7^.

PARP1 is a modular protein composed of six domains. Zinc-binding domains Zn1 and Zn2 recognize and bind to the DNA break (also referred to as zinc finger domains FI and FII;^8,9^. A third zinc-binding domain (Zn3, or FIII) and the Trp-Gly-Arg (WGR) domain contribute to DNA binding and support interdomain contacts that transmit the DNA binding signal to the catalytic domain (CAT)^10^. The CAT domain is composed of two subdomains: the helical domain (HD) and the ADP-ribosyl transferase domain (ART). The HD regulates PARP1 activity by blocking NAD^+^ binding to the active site^11^. A BRCT domain is located adjacent to an extended linker region that bears the primary sites for PARP1 auto-modification^12,13^. When PARP1 binds at the DNA damage site, the assembly of domains and their interactions on DNA promote partial unfolding of the HD allowing access of NAD^+^ to the catalytic site^10,11,14^. PARP2 and PARP3 domain structures are simpler, consisting of only a WGR and a CAT domain with short unstructured regions at their N-termini^15–17^. The HD substrate-blocking mechanism, which is relieved by DNA binding, is conserved in PARP2 and PARP3^11,14^.

Histone PARylation Factor 1 (HPF1) is a central regulator of PARP1 and PARP2 activity in the DNA damage response^18^. Indeed, HPF1 switches the amino acid specificity of PARP1 and PARP2 modifications from Glu/Asp to Ser ADP-ribosylation^18^. HPF1 was also shown to reduce PARP1 catalytic output, to decrease the length of PAR formed by PARP1 and PARP2, and to promote *trans* ADP-ribosylation of histones in relation to *cis* modification of PARP1 itself^19^. Crystal structures of HPF1 bound to the PARP2 CAT domain lacking an HD^20^, or to the PARP1 CAT domain lacking an HD^21^, have revealed the mechanistic basis for HPF1 effect on PARP1 and PARP2. HPF1 binds to PARP1 and PARP2 CAT and inserts a Glu residue to complement the active site (Fig. 1b). The HD limits HPF1 binding; therefore, unfolding of the HD through PARP1/2 interaction with DNA (or deleting the HD), is a pre-requisite for the most stable interaction with HPF1. The HPF1 interaction with PARP1/2 is further stabilized by compounds that maintain the HD in an open conformation, such as the NAD^+^ mimic known as EB47^20^. By comparison to the active site of cholera toxin-like ADP-ribosyl transferases, PARP1 and PARP2 are deemed to lack a second Glu residue, in addition to the catalytic Glu of the conserved His-Tyr-Glu triad motif (Glu988 in PARP1 and Glu545 in PARP2)^20^. The second Glu is provided by HPF1 and deprotonates Ser residues to allow their ADP-ribosylation^20,21^. In contrast to Ser, Glu and Asp are naturally deprotonated at neutral pH and therefore do not require HPF1 to be ADP-ribosylated by PARP1. In further support, a recent cryo-electron microscopy (EM) structure of a PARP2-HPF1 complex bound to nucleosomes provided a snapshot of how PARP2 assembles with HPF1 on a DNA break in the context of chromatin^22^. In each of the HPF1 complexes with PARP1/2, HPF1 sterically blocks the elongation or “acceptor” sites, thus indicating why HPF1 has a strong influence on PARP1/2 catalytic output and the distribution of PAR lengths produced.

Structural analysis has highlighted a 1:1 complex of HPF1 with PARP1/2 ready to perform ADP-ribose modification of Ser^20–22^. Moreover, PARP1 modification of Ser residues is the predominant form of PARylation performed by PARP1 in response to DNA damage^23^. However, HPF1 is about 20-fold less abundant than PARP1 in the cell^18,24^, raising the question of how HPF1 could function efficiently to support Ser modification by PARP1. Our study reveals that HPF1, in fact, can work efficiently to support Ser modification over Glu/Asp modification even at sub-stoichiometric ratios relative to PARP1, and the regulation is aided by a dynamic interaction between HPF1 and PARP1 as indicated by surface plasmon resonance (SPR) analysis of the interaction. We find that HPF1 regulates PARP1 and PARP2 activity not only by blocking the “acceptor” site and restricting PAR elongation, but also by markedly stimulating the rate of initiation of ADP-ribosylation, such that it is more comparable to the rate of elongation. HPF1 thus regulates the balance between initiation and elongation of the ADP-ribose modification. We also present hydrogen/deuterium exchange mass spectrometry (HXMS) data that support the dynamic PARP1/HPF1 interaction and indicate that HPF1 can further push the HD to the unfolded conformation induced by DNA damage binding, thereby contributing to PARP1 persistence on DNA damage. Negative-stain EM analysis of the PARP1-HPF1 complex bound to a DNA single-strand break provide first insights into the overall assembly of this dynamic complex. Our results support a model where HPF1 permits PARP1/2 initiation on Ser residues at a rate that greatly exceeds the rate of initiation on Glu/Asp residues, dynamically engages multiple PARP1/2 molecules before Glu/Asp initiation events occur, and suppresses elongation and thereby increases the opportunities for further initiation events. Together, our results and model explain how HPF1 efficiently supports modification of Ser residues by PARP1 in the cell despite its low abundance compared to PARP1, and they provide new understanding of the dynamic regulation of ADP-ribosylation in response to DNA damage.

## Results

### HPF1 efficiently regulates PARP1/PARP2 at sub-stochiometric ratios

HPF1 adapts the catalytic output of PARP1 and PARP2 such that ADP-ribose is linked to Ser residues rather than Glu/Asp residues^18^. The switching effect can be easily visualized by sodium dodecyl sulphate–polyacrylamide gel electrophoresis (SDS-PAGE) analysis where covalent PAR attachment causes a pronounced shift in PARP1/2 protein migration (Fig. 1c). Whereas the ester bond of PAR linked to Glu/Asp residues is sensitive to hydroxylamine treatment and thus reverses the PARP1/2 migration shift, the ether bond of PAR linked to Ser residues, due to the presence of HPF1, is resistant to hydroxylamine treatment (Fig. 1c)^18^. In contrast, PARP3 is not regulated by HPF1 and does not exhibit changes in hydroxylamine sensitivity (Supplementary Fig. 1)^18^. Prior to the structures of HPF1 bound to PARP1/PARP2^20–22^, we looked for structural features that might underlie the specificity toward PARP1/2, and we noted that PARP1/2 shared two features relative to PARP3 that localized to a similar region of the protein surface: a conserved Trp residue at the extreme C-terminus (PARP1–Trp1014; PARP2–Trp586), and a nearby loop containing a conserved His residue (PARP1–His826; PARP2–His381) (Fig. 1a, b). PARP1 bearing a three amino acid C-terminal deletion that removes Trp1014 (PARP1ΔCterm) was less responsive to HPF1, but otherwise showed wild-type levels of PAR production (Fig. 1d). Likewise, a charge reversal mutant of PARP1 at position His826 (H826E) was resistant to the effects of HPF1 on PAR linkage. His826 contributes to a region of PARP1 that regulates the PAR elongation reaction^3^ (Fig. 1a), and the mutant H826E indeed exhibits deficiencies in PAR production, making polymers of short sizes as evidenced by a reduced migration shift on SDS-PAGE (Fig. 1d). Regardless, the PAR produced by PARP1 H826E is Glu/Asp-linked in both the presence and absence of HPF1. Recent NMR, crystallographic, cryo-EM and mutagenic analyses of PARP1/2 interaction with HPF1 demonstrated that HPF1 indeed engages these distinct features of PARP1 and PARP2^20–22^. Our results are thus consistent with these structural snapshots, and the PARP1 mutants that we generated serve as controls for modulating the HPF1 interaction.

**Fig. 2:**
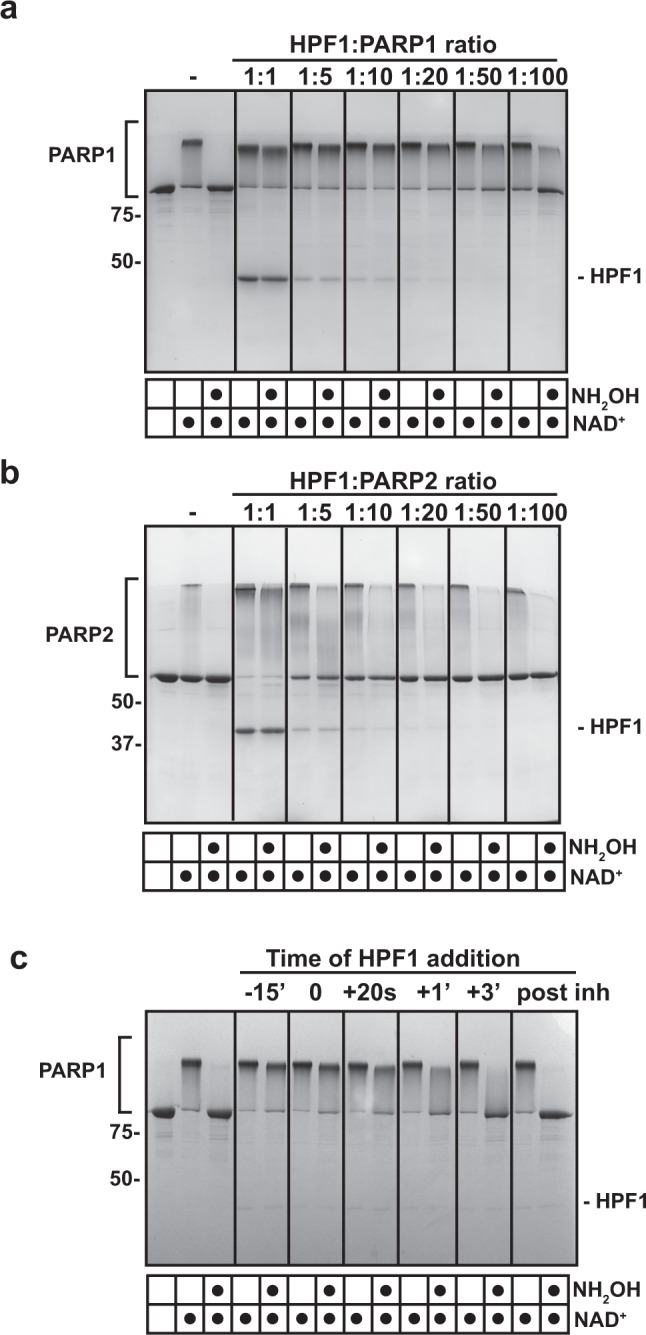
HPF1 works efficiently at sub-stochiometric ratios relative to PARP1 and PARP2. **a** PARP1 (1 μM) or **b** PARP2 (1 μM) was incubated with various amounts of HPF1 at the ratios indicated in the presence of DNA (1 μM) for 10 min at RT. Five-hundred micromolar NAD^+^ was added for 5 min and reactions were quenched with 500 μM PARPi (olaparib or talazoparib). Where indicated, reactions were treated with hydroxylamine (NH_2_OH) for 1 h. Reactions were resolved by SDS-PAGE and treated with Imperial Stain. **c** PARP1 (1 μM) was mixed with HPF1 (1 μM) at various time points relative to NAD^+^ addition (500 μM). For the −15’ reaction, HPF1 was incubated with PARP1 for 15 min prior to NAD^+^ addition. For the 0 reaction, HPF1 was added at the same time as NAD^+^. For the + reactions, HPF1 was added after NAD^+^ at the time indicated (20 s, 1 min, 3 min). For “post inh”, HPF1 was added after the reaction was quenched with PARP inhibitor. Reactions were then treated as in panels **a** and **b**. Experiments in **a**, **b**, and **c** were performed three times. Numbers on the left side of the gels represent molecular weight marker locations (values in kDa). Source data are provided as a Source Data file.

In our biochemical analysis of HPF1 control of PARP1 catalytic output, we noted that HPF1 could efficiently regulate the PARP1 specificity switch to Ser residues at sub-stoichiometric ratios, maintaining robust levels of Ser-linked modification even at HPF1 concentrations 10- to 20-fold lower than PARP1 (Figs.   1d and2a). HPF1 was indeed reported to be approximately 20-fold less abundant than PARP1 in the cell^18,24^, thus we considered that HPF1 could be tailored to biochemically operate at these sub-stoichiometric conditions. Even HPF1:PARP1 ratios of 1:50 and 1:100 yielded substantial amounts of Ser-linked modification. Similar results were also obtained for PARP2 (Fig. 2b), where PAR resistant to hydroxylamine treatment (i.e., Ser-linked) was observed even at the HPF1:PARP2 ratio of 1:100. However, the HPF1:PARP2 ratio of 1:1 was more efficient than the sub-stochiometric ratios at generating Ser-linked PAR. We first considered that HPF1 might impart a structural change on PARP1 that persists over time and thus does not require HPF1 to be continually engaged on each PARP1 molecule in order for Ser modification to occur. However, we were not able to isolate this putative, modified version of PARP1. Moreover, the recent structural analysis indicated that HPF1 directly contributes residue Glu284 to the PARP1/2 active site, thus indicating the requirement for a HPF1:PARP1/2 stoichiometry of 1:1 at the time of forming a Ser-linked ADP-ribose modification. We thus pursued a model in which HPF1 acts rapidly to modulate the output of multiple PARP1 molecules, moving from one molecule to the next to initiate Ser-linked modifications. In this model, HPF1 must act efficiently to engage consecutive PARP1 molecules, such that an excess pool of active PARP1 molecules is Ser-linked prior to the formation of Glu/Asp-linked modifications. Remarkably, we observed that HPF1 could efficiently modulate PARP1 output even when added at the same time as substrate NAD^+^ and at a HPF1:PARP1 ratio of 1:10 (Fig. 2c), indicating that HPF1 can rapidly engage PARP1, and also suggesting that initiation on Glu/Asp residues is a relatively slow process. We also considered the possibility that only a fraction of PARP1 molecules would be bound to DNA and active at any given time, as another potential explanation for efficient activity at the sub-stoichiometric HPF1:PARP1 ratios. However, HPF1 was also capable of regulating at a sub-stoichiometric ratio the constitutively active version of PARP1, PARP1ΔHD, which is active in the absence of DNA (Supplementary Fig. 2). Though it is possible that a certain number of Glu/Asp modifications are present along with Ser modifications on PARP1 molecules when HPF1 is present at sub-stochiometric levels, the majority of the modifications would have to be on Ser to explain the hydroxylamine protection results.

### Binding kinetics of HPF1 interaction with PARP1

**Fig. 3:**
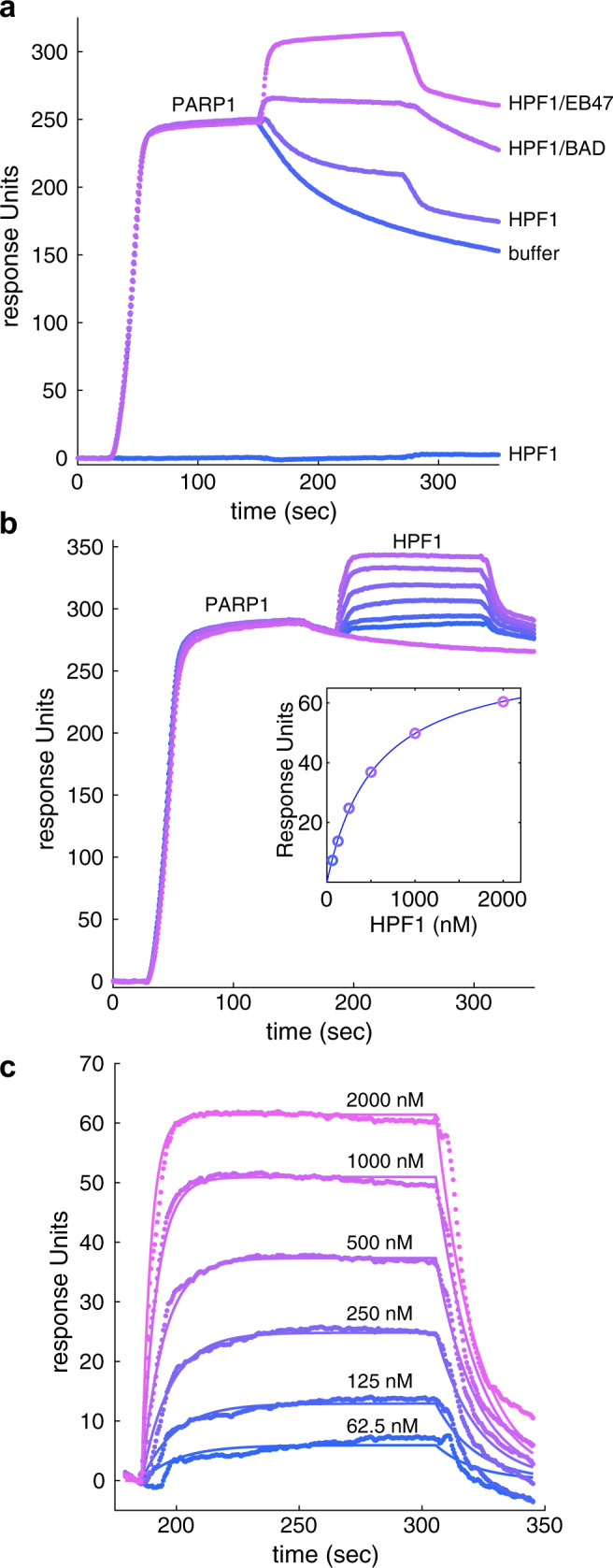
Kinetics of HPF1 interaction with the PARP1/DNA complex. **a** A DNA SSB was immobilized on a biosensor chip using streptavidin/biotin capture. PARP1 (40 nM) was flowed over the chip until near saturation, thus forming a PARP1/DNA complex. The surface was then immediately exposed to the following solutions: buffer, HPF1, HPF1 with BAD, or HPF1 with EB47. HPF1 alone did not interact with the DNA SSB surface. **b** HPF1 was passed over the PARP1/DNA complex at the following concentrations: 0 (buffer only), 62.5, 125, 250, 500, 1000, and 2000 nM. In these experiments, the buffer was supplemented with 5 µM EB47 to provide a more stable baseline during the PARP1 dissociation phase. The inset plots the plateau value achieved at each HPF1 concentration, and the line indicates the fit for a 1:1 binding model (*K*_D_ of 550 nM). **c** The association and dissociation phases of the HPF1 injections from panel **b** were fit to a 1:1 binding model, yielding a rate of association (*k*_a_) of 1 × 10^5 ^M^−1^second (s)^−1^ and a rate of dissociation (*k*_d_) of 0.06 s^−1^ (corresponding to *K*_D_ of 540 nM). These experiments were performed three times.

We evaluated HPF1 binding to PARP1 to gauge whether the kinetics of this interaction were consistent with the proposed model. Using SPR, we measured HPF1 interaction with PARP1 in complex with a biotinylated SSB DNA that was immobilized on a biosensor through streptavidin capture (Fig. 3). After PARP1 was allowed to associate with SSB DNA on the biosensor surface, HPF1 was injected during an early segment of the PARP1 dissociation phase, when PARP1 was still retained on the surface at high levels. HPF1 weakly interacted with the PARP1-DNA complex, but the interaction was greatly enhanced by the presence of the NAD^+^ mimic EB47, which supports the HD conformation that exposes the ART for optimal HPF1 binding (Fig. 3a). These results are consistent with a recent qualitative analysis of the HPF1-PARP1 interaction over gel filtration in the presence of DNA and EB47^20^. We observed a similar but less robust interaction when using the non-hydrolyzable NAD^+^ analog called BAD, which acts like EB47 to influence HD conformation, but has a lower binding affinity for PARP1 (Fig. 3a). It is notable that after the HPF1 dissociation phase, PARP1 was retained at a higher level on the surface relative to PARP1 that underwent dissociation in the presence of buffer instead of HPF1. We interpreted this observation to indicate that HPF1 had further stabilized PARP1 on DNA and thus slowed the rate of dissociation. As the PARP1-EB47 complex represents the active form of PARP1 and was the most stable species, we quantified HPF1 interaction with PARP1-DNA by performing SPR analysis with EB47 in the system buffer. The presence of EB47 also had the effect of stabilizing the baseline of PARP1 interaction with DNA during the dissociation phase. The steady state levels of HPF1 binding at different concentrations indicated a binding affinity of 480 ± 105 nM (Fig. 3b, c). In comparison, the binding affinity of HPF1 for PARP1-DNA (no EB47), or the ART domain alone (deleted HD), were reported as 3.5 µM and 1.5 µM, respectively^21,25^ and binding of HPF1 to a PARP2/nucleosome complex was reported as 280 nM^26^. We also analyzed the kinetics of the interaction. HPF1 exhibited a rate of association (*k*_a_) of 1.12 ± 0.14 × 10^5^ M^−1^s^−1^ and a rate of dissociation (*k*_d_) of 0.071 ± 0.033 s^−1^ (*K*_D_ of 617 ± 213 nM). The *k*_d_ of 0.071 s^−1^ corresponds to a half-life of roughly 10 s. We reversed the SPR setup and attached HPF1 to the biosensor using amine coupling, and observed similar results regarding the requirement of DNA and an NAD^+^ mimic for robust interaction, and we also verified that the H826E mutant abolished the HPF1-PARP1 interaction (Supplementary Fig. 3). Overall, the SPR analysis indicated that HPF1 interaction with PARP1 is “hit and run” with rapid association and dissociation, rather than an extended dwell time with PARP1 in the activated state. The results are consistent with our model where one HPF1 molecule visits several PARP1 molecules and participates in the catalysis of Ser ADP-ribosylation before PARP1 performs modification of Glu/Asp residues.

### HPF1 stimulates initiation and restricts elongation

**Fig. 4:**
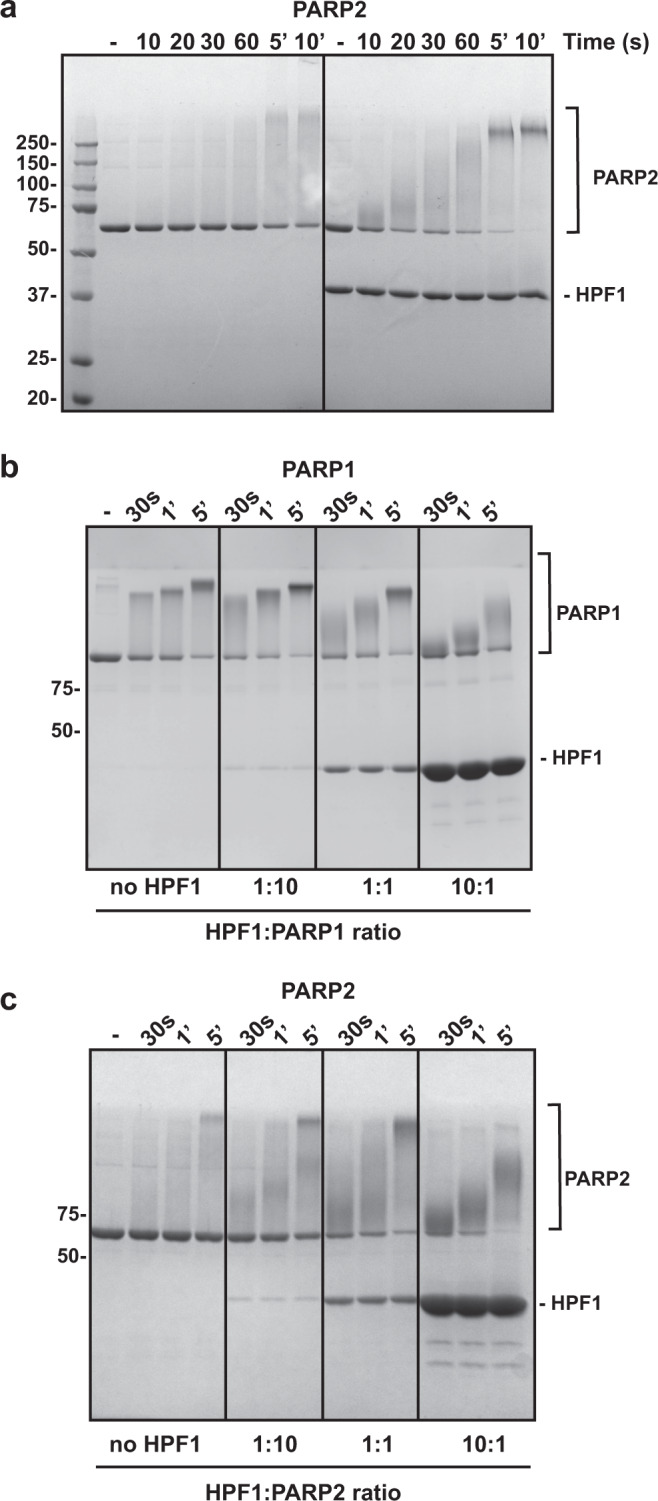
HPF1 restricts elongation and stimulates initiation in PARP2. **a** PARP2 (1 μM) was incubated with HPF1 (1 μM) for 10 min at RT in the presence of DNA (1 μM), where indicated. 500 μM NAD^+^ was added for various time points (except in the “-” reaction), and reactions were quenched with SDS-PAGE loading buffer, resolved by SDS-PAGE, and treated with Imperial stain. **b** PARP1 (1 μM) was incubated with HPF1 at various ratios as indicated in the presence of DNA (1 μM) for various time points with 500 μM NAD^+^. Reactions were processed as in panel **a**. **c** Same as in panel **b** for PARP2. Experiments in Fig. 4 were performed two times. Numbers on the left side of the gels represent molecular weight marker locations (values in kDa). Source data are provided as a Source Data file.

**Fig. 5:**
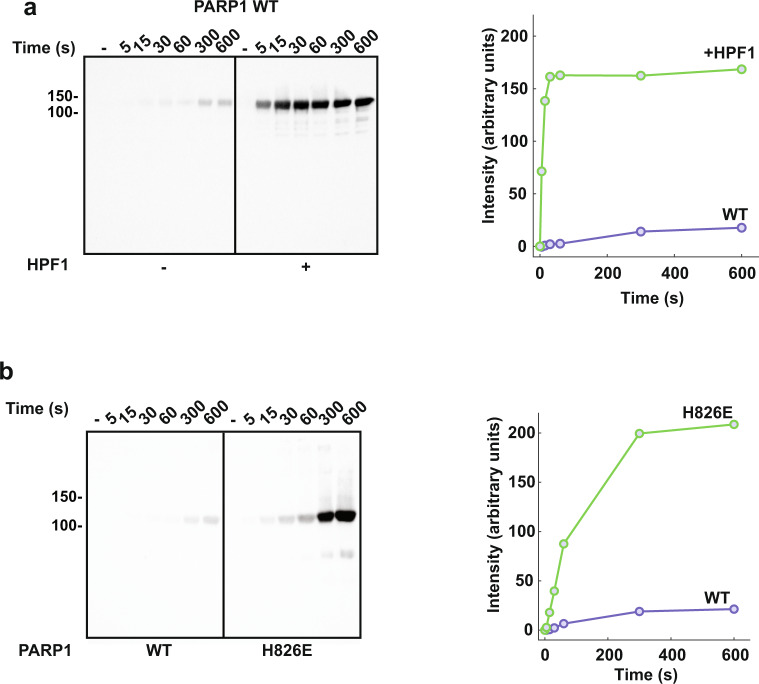
HPF1 stimulates initiation in PARP1. **a** PARP1 (1 μM) was incubated without or with HPF1 (0.1 μM) for 10 min at RT in the presence of DNA (1 μM). 1 mM NAD^+^ was added for various time points as indicated, and reactions were quenched with 500 μM talazoparib. PARG (1 μM) was added and incubated for 1 h at RT. Reactions were resolved by 12% SDS-PAGE and a western blot was performed using a mono-ADP-ribose-binding reagent. The bands corresponding to mono-ADP-ribosylated PARP1 were quantified using ImageJ and plotted as intensity over time. **b** Reactions were performed as in panel **a** without HPF1 and comparing PARP1 WT to PARP1 H826E. Reactions were resolved by 7.5% SDS-PAGE and a western blot was performed using a mono-ADP-ribose binding reagent. For 5a and b, a representative image from three independent experiments is shown. Numbers on the left side of the blots represent molecular weight marker locations (values in kDa). Source data are provided as a Source Data file.

Our model also predicts that the HPF1-dependent Ser modification is faster than Glu/Asp modification. We observed indeed a stimulation of PARP2 initiation events in the presence of HPF1, visualized by a rapid decrease in intensity of the unmodified PARP2 band as early as 10 s in the presence of HPF1 (Fig. 4a). In contrast, a similar decrease in unmodified band intensity is only observed after 5 min (min) in the absence of HPF1 (Fig. 4a). In the case of PARP1, HPF1 stimulation of catalysis is not clearly observed when considering global activity on SDS-PAGE (Fig. 4b). In fact, we noted that HPF1 limits the length of polymer produced by PARP1, as previously described^19^. HPF1 inhibition of PAR growth is more and more pronounced as the concentration of HPF1 was increased in PARP1 and PARP2 activity assays, indicating that HPF1 has the capacity to severely restrict elongation when used at elevated concentrations (Fig. 4b, c). This restriction is easily explained by the fact that HPF1 binding blocks access to the ADP-ribose acceptor site^20–22^. To more directly focus on the HPF1 effect on PARP1 initiation reactions (as opposed to both initiation and elongation), we quantified PARP1 initiation rates in the presence and absence of HPF1. To accomplish this analysis, we performed ADP-ribosylation reactions for various time points and treated the quenched reactions with poly(ADP-ribose) glycohydrolase (PARG), an enzyme that digests the PAR polymer but leaves the initial ADP-ribose attached to PARP1^27^. The resulting mono(ADP-ribose) modifications on PARP1 were detected in a Western blot using a binding reagent that recognizes mono(ADP-ribose)^28^. Strikingly, we observed in the presence of HPF1 that initiation was near saturation at 30 s, whereas initiation in the absence of HPF1 did not reach saturation until greater than 300 s (Fig. 5a and Supplementary Fig. 4A). Using the early time-points in the linear region to estimate a rate of reaction, we observed that HPF1 increases the rate of initiation by 40-fold relative to PARP1 alone (Supplementary Fig. 5A, B, C). The initiation rate that we measured in the absence of HPF1 (0.0033 s^−1^ ± 0.0015; Supplementary Fig. 5A, C) is comparable to the *k*_cat_ measured previously (0.0055 s^−1^) using 3’ deoxy-NAD^+^, which is limited to the initiation reaction since the ADP-ribose cannot be extended^29^. In the presence of HPF1, the initiation rate was increased to 0.12 s^−1^ ± 0.02 (Supplementary Fig. 5B, C). As a comparison, the rate of the global PARP1 PARylation reaction (which is likely to represent primarily elongation) was reported to be between 0.3 and 5.2 s^−1^ ^3,25,30–33^. Interestingly, the signal plateau reached by PARP1 in the presence of HPF1 is higher than the plateau reached in the absence of HPF1 (Fig. 5a, compare 300 s and 600 s with and without HPF1). Altogether, the data indicate that more initiation events take place in the presence of HPF1, and that the rate of accumulating initiation events is more efficient in the presence of HPF1.

We considered that the HPF1 block on the PARP1 elongation site could in effect divert the PARP1 active site to carry out more initiation events, thus explaining the observed increase in maximum signal. To test this reasoning, we used the PARP1 mutant H826E as a mimic of HPF1 disruption of the elongation site. As previously noted, H826E produces shorter polymers than WT due to the fact that His826 forms part of the ADP-ribose acceptor site (Fig. 1a, d). The rate of initiation for the H826E mutant is about 20-fold higher than WT PARP1 (Fig. 5b and Supplementary Figs. 4B and 5D), and the maximum signal for this mutant was also higher than WT. The addition of HPF1 did not affect the activity of H826E (Supplementary Fig. 6), consistent with the fact that HPF1 does not bind to this PARP1 mutant (Supplementary Fig. 3)^20^. Though the maximum signal of H826E was higher than WT PARP1, it did not reach the level observed for WT PARP1 in the presence of HPF1 (Supplementary Fig. 6). Together, these results suggest that restricting elongation indeed has the consequence of diverting PARP1 activity toward initiation events, and this mode of regulation can be invoked by HPF1 or through mutagenesis. However, the HPF1 effect on initiation is not due solely to restriction of elongation, since HPF1 substantially stimulates the rate at which PARP1 reaches saturation in our analysis of initiation sites.

HPF1 also modulates PARP1 catalytic output by increasing the extent of trans-modification of other factors, such as histones, relative to auto-modification^19^. For example, histone H3 is not appreciably trans-modified by PARP1 unless HPF1 is present, and there is a substantial decrease in PARP1 auto-modification in the presence of HPF1 and histone H3 or histone octamer^18,19^. To determine if HPF1 affects PARP1 trans-ADP-ribosylation at sub-stochiometric ratios at the initiation step, we incubated PARP1 with histone octamer in the presence and absence of HPF1, and treated the quenched reaction with PARG (Supplementary Fig. 5E and Supplementary Fig. 4C). Notably, the histone octamer inhibited PARP1 auto-modification even in the absence of HPF1. In the presence of HPF1 at a 1:10 ratio, PARP1 auto-modification was greatly reduced and one or more histones were modified indicating that even at sub-stochiometric ratio, HPF1 is able to modulate PARP1 catalytic output from auto- to trans-modification.

### HPF1 destabilizes the PARP1 HD

**Fig. 6:**
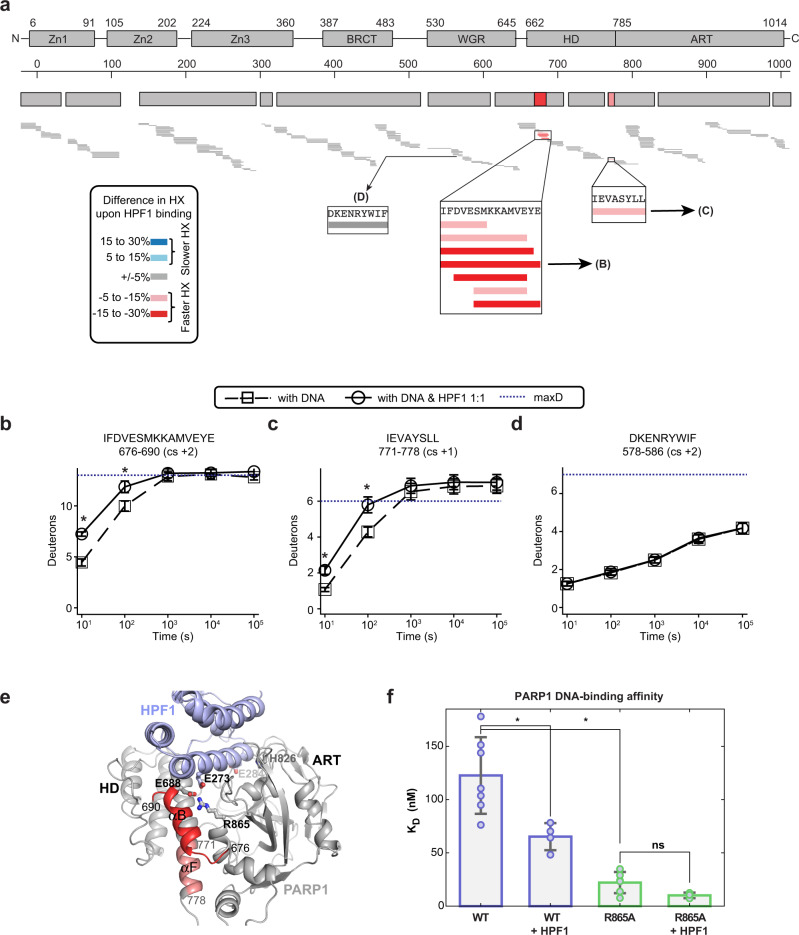
HPF1 further destabilizes the HD when PARP1 is bound to a DNA SSB. **a** An HXMS difference plot obtained by subtracting the percent deuteration of the PARP1/DNA/HPF1 complex (at 1:1 ratio of HPF1:PARP1) from PARP-1/DNA complex at 10 s. Each horizontal bar represents a PARP1 peptide. Most of the peptides (gray) in the PARP1/DNA/HPF1 complex have similar HX rates when compared to the PARP1/DNA complex. However, peptides in the αB and αF helices of HD showed faster exchange (red). The white regions in the difference plot represent gaps in the peptide coverage. **b** HX of a representative peptide from the αB helix in panel **a** for PARP1/DNA and PARP1/DNA/HPF1. **c** HX of a representative peptide from the αF helix in panel **a** for PARP1/DNA and PARP1/DNA/HPF1. **d** HX of representative peptide from WGR domain in panel **a** for PARP1/DNA and PARP1/DNA/HPF1. In panels **b** to **d**, an average is shown with error bars representing SD and asterisks indicating *P* < 0.05 (*t*-test two-sided between PARP1/DNA and PARP1/DNA/HPF1). For αB at 10 s, *P* = 0.0003, at 100 s, *P* = 0.0121. For αF at 10 s, *P* = 0.0018, at 100 s, *P* = 0.0073. Experiments were performed in triplicate. In panels **b**–**d**, the blue dotted lines indicate the maximum number of exchangeable deuterons (maxD). **e** Consensus HXMS data from panel **a** mapped onto the structure of the catalytic domain of PARP1 (from 4DQY), which was modeled in complex with HPF1, based on the PARP1CATΔHD complex with HPF1 (6TX3). **f** DNA binding affinity of PARP1 in the absence and presence of HPF1 determined by fluorescence polarization. An average of six independent experiments for PARP1 WT and PARP1 R865A, four independent experiments for PARP1 WT with HPF1, and three for PARP1 R865A with HPF1 is shown with associated SD represented by error bars. Two-sample two-sided *t*-tests were used to compare the *K*_D_ values and asterisks indicate *P* < 0.05. ns, indicates not significant. The *P*-values are 0.014 for WT and WT + HPF1, 0.000039 for WT and R865A, and 0.087 for R865A and PARP1 R865A + HPF1. Source data are provided as a Source Data file.

We undertook HXMS analysis to gain insights into HPF1 influence on PARP1 dynamics, in particular the dynamics of the HD and DNA binding regions. HXMS monitors the exchange of backbone amide hydrogens of a protein and thereby reports on protein structure and dynamics. Using this technique, we previously identified PARP1 regions that exhibit dramatic increases in HX, as well as regions that exhibit decreases in HX, when PARP1 is bound to DNA^14^. The increases in HX locate to specific HD helices and relate to the allosteric mechanism that permits NAD^+^ binding, and the decreases in exchange locate to PARP1 regions that directly engage DNA or form domain-domain contacts. More recently, we used HXMS to study the effect of NAD^+^ analogs and PARP inhibitors on the dynamics of the PARP1 complex with DNA^11,34^. Here, PARP1 was assembled on a DNA structure that models a DNA SSB, and the effect of adding HPF1 was analyzed through a full time-course HXMS experiment (from 10^1^ to 10^5^ s). Comparing HXMS data at 10 s of the PARP1/HPF1/DNA complex to the PARP1/DNA complex, we observed an increase in HX for the peptides in helix B (676- 690) and a peptide at the C-terminus of helix F (771-778) of the HD (Fig. 6a–d and Supplementary Fig. 7). Notably, differences in HX rates of the PARP1/DNA/HPF1 complex when compared to PARP1/DNA complex were only seen at 10 s and 100 s (see 100 s timepoint in Supplementary Fig. 8). Helix B and helix F are the very same helices that partially unfold when PARP1 is bound to a DNA break^14^. Moreover, the PARP2/HPF2/nucleosome cryo-EM structure indicated structural rearrangements and dynamic states for helix B and helix F when compared to crystallographic structures of PARP1 and PARP2 in the absence of HPF1^22^, consistent with our results. Our results indicate that HPF1 pushes the HD further toward the unfolded state, with the potential to influence PARP1 access to substrate NAD^+^ and thus catalytic output.

In contrast to NAD^+^ mimics, HPF1 did not induce an increase in protection in PARP1 regions directly involved in DNA binding. However, HPF1 did increase PARP1 affinity for DNA by about 2-fold in a fluorescence polarization DNA binding experiment (Fig. 6f). These results are consistent with the SPR results where the addition of HPF1 increased PARP1 retention on DNA (Fig. 3a). These data suggest that HPF1 destabilization of the HD does exert an allosteric effect on PARP1 binding to DNA, similar to the binding of Type I inhibitors such as EB47^34^, the NAD^+^ analog BAD^11^, and the veliparib derivative UKTT15^34^, albeit to a lesser extent. The structure of PARP1 CAT ΔHD bound to HPF1 shows that PARP1 residue Arg865 is repositioned to interact with HPF1 residue Glu273 when compared to the structure of PARP1 CAT without HPF1, in which Arg865 interacts with HD residue Glu688 located on helix B (Fig. 6e). Breaking the connection between the HD and the ART by HPF1 could promote HD conformational flexibility and thereby increase PARP1 retention on DNA. To investigate this further, we created the PARP1 R865A mutant. The R865A mutation did not disrupt the ability of PARP1 to interact with HPF1 since the R865A mutant was still able to modify Ser in the presence of HPF1 (Supplementary Fig. 9). However, the R865A mutant showed a 4-fold increase in affinity for DNA breaks compared to WT (Fig. 6f). The addition of HPF1 to R865A did not further increase the affinity of PARP1 R865A for DNA, in contrast to what was observed for PARP1 WT, suggesting that the R865A mimics the effect of HPF1 on the HD/ART connection and consequently on PARP1 affinity for DNA.

The findings of a transient interaction with PARP1 and a massive stimulation in the PAR-chain initiation step, along with the prior report that the HD must be removed to allow stable binding^20^, suggest a model where the HD flexibility first permits HPF1 to recognize only PARP1 molecules engaged with a DNA break, but then stabilizes PARP1 on the break long enough (seconds timescale) to initiate PAR chains on local target serine residues. The idea of HPF1 stabilizing PARP1 on a DNA break initially seems at odds with the finding that genetic removal of HPF1 leads to PARP1 retention for several minutes on large lesions of DNA damage^19^. The answer to this paradox may lie in a role for serine-linked PARylation in auto-modification-dependent release from these lesions that is defective in the absence of HPF1. A recent publication indeed has shown that the three main PARP1 Ser residues modified in the presence of HPF1 are essential for proper PARP1 release from DNA^35^.

### Negative-stain EM analysis of the PARP1/HPF1/DNA complex

**Fig. 7:**
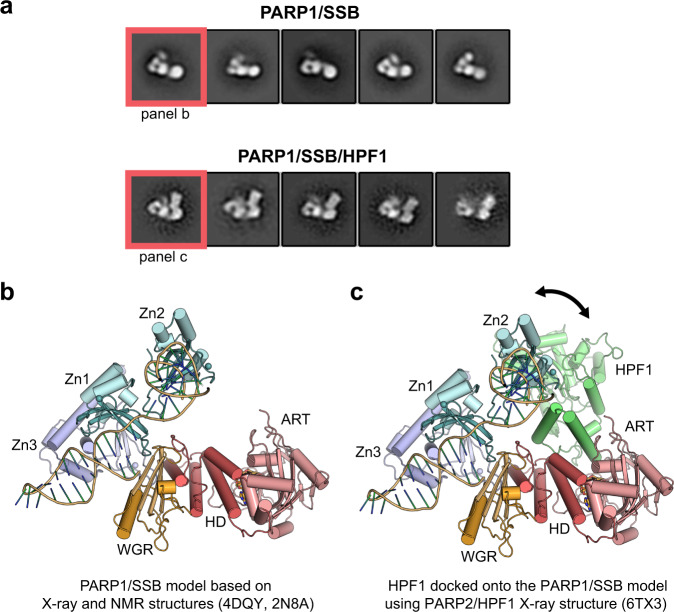
Architecture of the HPF1 complex with full-length PARP1 bound to a DNA break. The PARP1/DNA SSB complex was analyzed by negative-stain electron microscopy in the absence and presence of HPF1. **a** 2D class averages provided views of the complexes without and with HPF1 that could be interpreted with the aid of published structures of PARP1 domains (Zn1, Zn2, Zn3, WGR, and CAT (HD/ART)) on DNA as shown in **b** and **c**. **b** A combined model of a PARP1 Zn1/Zn3/WGR-CAT/DNA structure (4DQY) aligned to a PARP1 Zn1/Zn2/DNA NMR structure (2N8A) **c** Same as in **b** with HPF1 added based on the structure of PARP2 CATΔHD/HPF1 (6TX3)^43^. The 2D averages did not suggest a location for the BRCT domain, which we infer to remain flexibly tethered to the rest of the complex. Consistent with our interpretation of PARP1 domains, the addition of HPF1 adds to the globular end of the protein interpreted as the ART (panel **c**). The orientation of HPF1 with respect to the catalytic domain remains relatively fixed, whereas the orientation of HPF1 relative to the PARP1 regulatory domains appears to adopt two different conformations, highlighted by the arrow in panel **c**.

Structural insights into full-length PARP1 interaction with HPF1 are still lacking. Therefore, we used negative-stain EM to provide first views of a PARP1-HPF1-DNA complex. PARP1 was incubated with a DNA SSB containing a one-nucleotide gap in the presence of EB47, both with and without HPF1. The EM analysis indicates that PARP1 binds to SSB-DNA as a monomer in the absence and presence of HPF1 (Fig. 7a). Overall, the 2D classification indicated that PARP1 forms a largely globular assembly, yet there is inherent flexibility within the assembly that prevented the construction of confident 3D maps. The DNA was not clearly visible in the images, likely due to the DNA size and multiple PARP1 domains enveloping the DNA structure. The 2D class averages provided views in which certain PARP1 domains could be inferred based on knowledge from X-ray and NMR structures of PARP1 fragments (composite X-ray/NMR models are shown in Fig. 7b, c for comparison to 2D class averages in Fig. 7a). The domains evident in the 2D class averages are expected to represent the most stable components of the complex: the Zn1, Zn3, WGR and CAT domains. The 2D averages did not suggest a location for the BRCT domain, which we infer to remain flexibly tethered to the rest of the complex. Consistent with our interpretation of PARP1 domains and their relative arrangement, additional density was observed on the CAT domain in the presence of HPF1. In the 2D averages, there appear to be two main conformations for the PARP1-HPF1-DNA complex, one where HPF1 is flexed away from the domains assembled on DNA, and one where HPF1 approaches these domains (Fig. 7a, c). The negative-stain EM imaging thus indicates that HPF1 is incorporated into the PARP1 assembly of domains on a DNA break, with the potential to influence the overall stability of the PARP1-DNA complex. Moreover, HPF1 is positioned within the complex in a way that could influence PARP1 capacity to automodify, and therefore play a role in the regulation of *cis versus trans* modification.

## Discussion

**Fig. 8:**
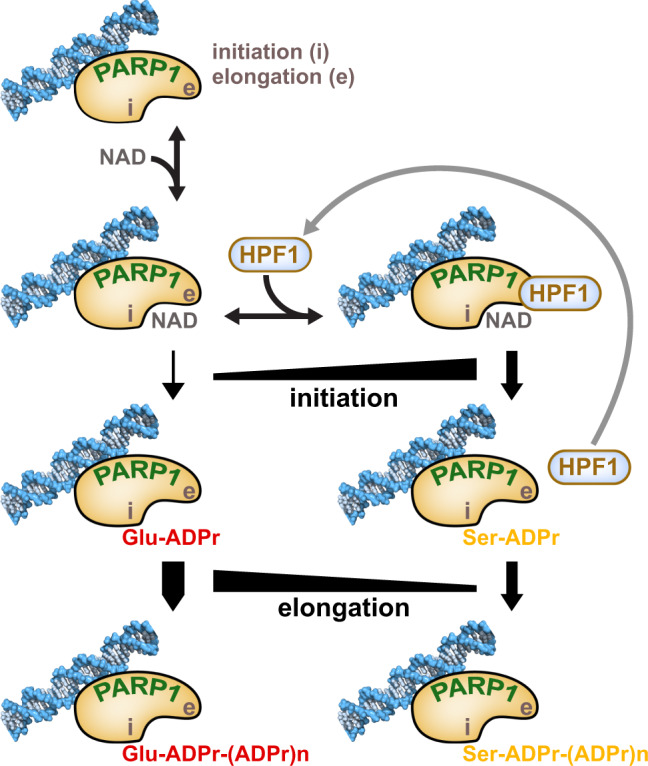
Model for HPF1 regulation of PARP1/2 catalytic output. HPF1 regulates the balance between initiation (i) and elongation (e) through a dynamic interaction with the catalytic domain and by accelerating the rate of initiation on Ser residues. Rapid HPF1 cycling between PARP1 molecules, indicated by the long arrow, allows the PARP1 population to be efficiently modified on Ser, and also prevents excessive inhibition of the elongation reaction to form PAR that is important for DNA damage signaling.

The recent determination of high-resolution structures of PARP1 and PARP2 bound to HPF1^20–22^ have had a tremendous impact on our comprehension of HPF1 mode of action. HPF1 completes the active site of PARP1 by inserting catalytic residue Glu284, and this HPF1-PARP1 complex is essential for Ser ADP-ribosylation, the predominant type of modification in cells following DNA damage. It is thus tempting to imagine a stable and saturated HPF1-PARP1 complex to ensure that the appropriate target residues are modified. However, the strong inhibitory influence of HPF1 on the elongation reaction complicates this picture, and the cellular abundance of HPF1 does not match that of PARP1. The results presented in this study allow us to propose a new model for HPF1 regulation of PARP1/2 catalytic output (Fig. 8). Our data suggest that HPF1 is bound to PARP1 at the step of initiation, where it substantially stimulates the rate of attachment of the first ADP-ribose to Ser in comparison to the rate observed for the attachment of ADP-ribose to Glu/Asp in the absence of HPF1. For the time that HPF1 remains bound to PARP1, the elongation reaction is inhibited through a steric block on the acceptor site. Yet, the PARP1 active site remains available for additional initiation reactions on Ser residues, explaining why we observe a higher number of initiation events in the presence of HPF1. This shift in balance toward initiation events was also observed with PARP1 mutant H826E, which has a disrupted acceptor site and is restricted in PAR elongation. These results suggest that the elongation site could serve as a general engagement site for factors (proteins or small molecule ligands) to bind and modulate PARP1/2 catalytic output.

Our results showing that HPF1 functions efficiently at sub-stoichiometric ratios indicate that HPF1 must visit and initiate Ser modification on multiple PARP1 molecules before PARylation is initiated on Glu/Asp residues, hence the “hit and run” mechanism that we propose. Our SPR data show that indeed HPF1 can associate/dissociate rapidly from PARP1. Therefore, we propose that there is a fine balance between HPF1 remaining bound to PARP1 and blocking PAR chain elongation, and HPF1 dissociating and binding to a different PARP1 molecule to ensure that ADP-ribosylation takes place on Ser residues.

It is notable that stochiometric amounts of HPF1, or an excess of HPF1, has the effect of strongly inhibiting global PARP1 and PARP2 PARylation activity (Fig. 4b, c), due to the steric blockage of the elongation/acceptor site, reducing more and more the size of the polymer as the concentration of HPF1 is increased. These results highlight that saturating the HPF1 interaction with PARP1 reduces catalytic output to a level that would be not be productive. In fact, a recent study has shown that in the presence of an excess of HPF1, PARP1 exhibits a preference to hydrolyze NAD^+^ instead of converting it to ADP-ribose chains, after preferred protein substrates are depleted^25^. These conditions of excess HPF1 are not expected to arise in the cell, but the striking consequences highlight the potency of HPF1 regulation. The relatively high *K*_D_ for the PARP1/HPF1 interaction (0.5 μM in this study with a DNA break, 3.5 μM in a different study in the absence of DNA^25^), combined with known cellular ratio of HPF1 to PARP1, suggests that PARP1 might be rarely saturated with HPF1, unless HPF1 concentrations vary substantially between various cell types.

The ratio of HPF1 to PARP1 in the cell could reflect a balance of ensuring efficient initiation of Ser modification, while also limiting the impact on ADP-ribose polymer production that is key to multiple aspects of DNA damage repair signaling. Our model also applies to trans-modification of histones; even at the HPF1:PARP1 ratio of 1:10, HPF1 supports the switch from auto-modification of PARP1 to trans-modification of histones as observed with the large decrease in signal observed on PARP1 in the presence of HPF1 and the histone octamer (Supplementary Fig. 5E). Although HPF1 was reported to be modified by PARP1 on Y238^36,37^, we did not observe evidence for ADP-ribosylation of HPF1 during the time-course of our assays, suggesting that HPF1 modification itself might not have a strong influence on the ability of HPF1 to cycle between many PARP1 molecules during the course of the reaction.

PARP1 initiation in the absence of HPF1 is rather inefficient (Fig. 5a). This observation is not readily apparent when observing the global activity on SDS-PAGE (e.g., Figs. 1c, 4b). Previous PARP1 studies without HPF1 have shown that the elongation step of PAR formation is more efficient than the initiation step^31,38,39^, and PAR size analysis from PARP1 reactions has indicated a distribution skewed toward long polymer sizes^40^, which supports slow initiation but rapid elongation events. Thus, in the absence of HPF1, initiation on Glu is a relatively slow and inefficient event, but once a Glu initiation event has occurred, the elongation step is much more rapid, creating long chains of PAR that shift the PARP1 molecule on SDS-PAGE. Therefore, globally, the PARylation reaction appears to be quite efficient in the absence of HPF1. In the presence HPF1, the balance of initiation *versus* elongation is shifted and the rate of initiation is substantially increased, such that the complete pool of PARP1 molecules is initiated within seconds. This effect can be clearly observed in our analysis of initiation events (Fig. 5), and it can also be appreciated by observing the loss of unmodified PARP1 in the SDS-PAGE reactions at early time-points (Fig. 4b). HPF1 thus elevates the rate of initiation substantially such that initiation is more in balance with elongation.

Interestingly, the poly- and mono-ADP-ribose levels in the cell were shown recently to be tightly controlled by the combined action of PARG, which degrades PAR chains, and ARH3, which removes the last mono-ADP-ribose on Ser, adding another level of complexity to the regulation of ADP-ribose production, where the initiation and elongation steps can be regulated^41^. Indeed, the efficient removal of PAR chains by PARG could effectively increase the contribution of the initiation reaction to the net cellular ADP-ribose level.

Stochiometric amounts of HPF1, or an excess of HPF1, has the effect of strongly inhibiting global PARP1 and PARP2 PARylation activity (Fig. 4b, c), due to the steric blockage of the elongation/acceptor site, reducing more and more the size of the polymer as the concentration of HPF1 is increased. HPF1 inhibition of elongation is likely to contribute to the shift toward initiation. The compact conformation of PARP1 on a DNA break was proposed to position residues for initiation of the ADP-ribose modification^10,42^. Ser residues and Glu residues identified consistently in mass spectrometry analysis of auto-modification sites^13,18,43,44^ are located in a region predicted to position near the active site of PARP1^10,42^. The Ser residues could be in the “most optimal” position for modification compared to Glu residues, and HPF1 simply allows these Ser residues to be utilized by completing the active site^20^. However, the automodified region represents a lengthy linker region that is likely to be quite flexible, thus it is challenging to envision a rigidly fixed, “most optimal” region to be modified. The HPF1-PARP2 crystal structure identified a cleft near the active site termed the “canyon” that was proposed to specifically engage peptides to be modified^20^. The peptide canyon could contribute to the acceleration of initiation events by providing a specific site of engagement for target residues, and a Lys-Ser motif is emerging as a signature sequence that could be engaged.

Overall, our study adds a new level of understanding of HPF1, and how it dynamically influences the initiation and elongation steps of PARP1/2 PARylation activity. These special properties allow HPF1 to regulate the balance of PARP1/2 catalytic output to match the requirements for ADP-ribose production in the cellular response to DNA damage, both in terms of the speed of catalyzing ADP-ribose modification and the size distribution of polymer formed. We also present HXMS and negative-stain EM analysis that provide first insights into the structure and dynamics of full-length PARP1 in complex with HPF1, providing a framework for better understanding the special collaboration between these proteins in the DNA damage response.

## Methods

### Expression constructs and mutagenesis

PARP1 (residues 1 to 1014) and PARP2 (isoform 2, residues 1 to 570) were expressed from a pET28 vector with an N-terminal hexahistidine tag. PARP3 FL (isoform b, residues 1 to 533) was expressed from a pDEST17 vector with an N-terminal hexahistidine tag (gift from Dr. Ivan Ahel, University of Oxford). The human HPF1 gene was synthesized for expression from a pET28 vector with an N-terminal SMT sumo-like His-tag. Human histones genes were synthesized for co-expression: H2A/H2B in vector pCDF Duet, and H3/H4 in vector pET Duet-1. Human PARG (residues 488-976) was expressed from a pET28 vector N-terminal hexahistidine tag (gift from Dr. Ivan Ahel, University of Oxford). Site-directed mutagenesis was performed using the QuikChange protocol (Stratagene) and verified by automated Sanger sequencing. PARP1 ΔHD was prepared using sortase-mediated joining of two PARP1 fragments (N-terminal fragment and C-terminal fragment). Sortase fragment 1 was produced from a pET24 construct coding for residues 1–375 and a C-terminal linker LPTEG (sortase recognition sequence). Sortase fragment 2 was produced from a pET28 construct coding for residues 383–703/779–1014 with an N-terminal SMT sumo-like His-tag that was removed during purification.

### Protein expression and purification

PARP1 WT and mutant proteins were expressed and purified as described previously using Ni^2+^-affinity, heparin, and gel filtration chromatography^8,14,45,46^. PARG was purified using Ni^2+^-affinity and gel filtration chromatography. Purification of PARP2 and PARP3 was performed as described^15^ using Ni^2+^-affinity, heparin, and gel filtration chromatography. Purification and reconstitution of PARP1 ΔHD was performed as described^14^ using the transpeptidase Sortase A. Sortase fragment 1 was purified using Ni^2+^-affinity and heparin columns. Sortase fragment 2 was purified using a first Ni^2+^-affinity column. The SMT sumo-like tag was then cut using ULP1 protease and the protein mixture was passed a second time over a Ni^2+^-affinity column, with cleaved Sortase fragment 2 in the flow-through due to the removed histidine/SMT tag. The fragment was then purified on a heparin column. The sortase reaction was performed using 245 μM of 383–1014, 510 μM 1–375 LPETG, and 5.4 μM sortase in a 500 μL reaction in 50 mM Tris pH 8.0, 50 μM ZnSO_4_, 1 mM DTT, 10 mM CaCl_2_, and 200 mM NaCl. The sortase reaction was dialyzed at 4 °C overnight, and then passed over a Ni^2+^-affinity column. The sortase reaction removed the His-tag from 1–375 LPETG, thus the FL ΔHD protein was found in the flow-through of the Ni^2+^ column, which was then further purified on a heparin column. Histones H2A and H2B were co-expressed in *E. coli* and purified as a soluble dimer using anion exchange, heparin, and gel filtration chromatography. Histones H3 and H4 were co-expressed in *E. coli* and purified as a soluble tetramer using anion exchange, heparin, and gel filtration chromatography. The histone octamer was formed by mixing H2A/H2B with H3/H4 and further purification was performed on a Sephacryl S200 gel filtration column. HPF1 was purified using a Ni^2+^-affinity column. The SMT sumo-like tag was then cut using ULP1 protease and the protein mixture was passed a second time over a Ni^2+^-affinity column, with cleaved HPF1 in the flow-through due to the removed histidine/SMT tag. The flow-through was then diluted to 50 mM heparin buffer and the rest of the purification was performed as described for PARP1^8,14,45,46^.

### SDS-PAGE PARP1 activity assay

The SDS-PAGE activity assay was performed as described^46^ using 1 μM PARP1 or PARP2, various amounts of HPF1 as indicated, 1 μM DNA (dumbbell with a central nick for PARP1: 5’GCT GAG CTT CTG GTG AAG CTC AGC TCG CGG CAG CTG GTG CTG CCG CGA; 28 bp duplex DNA with a 5’ Phosphate for PARP2: 5’ CGA GTC TAC AGC GTT GCG GCC GCT TGG G annealed to a complementary strand), and 500 μM NAD^+^. Reactions were incubated for 5 min or as indicated before stopping the reaction with PARP inhibitor veliparib, olaparib, or talazoparib at 500 μM. Where indicated, reactions were treated with 1 M hydroxylamine (NH_2_OH) for 1 h at room temperature, then quenched with 0.3% HCl. SDS-PAGE loading buffer was added to the reactions prior to resolution on a 12% SDS-PAGE, which was treated with Imperial stain for visualization.

### Binding analysis using SPR

SPR was performed on a Reichert 4SPR biosensor equipped with a supplementary valve that allows for sequential injections. The system buffer was 25 mM HEPES pH 7.4, 250 mM NaCl, 0.1 mM TCEP, 1 mM EDTA, and 0.05% Tween20. The buffer was supplemented with 5 µM EB47 for certain experiments as indicated. Streptavidin-coated chips (Reichert) were used to capture a DNA SSB bearing a biotin group (20 nM) (5’ GCT GAG CTT CTG GTG AAG CTC AGC TCG CGG CCT GGC GCC AGC BIOTGG TGC TGG CGC CAG GCC GCG). PARP1 was flowed over the biosensor at 40 nM, and HPF1 was then injected over a range of concentrations in the absence or presence of EB47 (5 µM) or BAD (300 µM). CM5 chips (Reichert) were used to immobilize HPF1 using standard amine coupling procedures. PARP1 (800 nM) was flowed over the HPF1-coated biosensor in the absence or presence of DNA (800 nM) and EB47 (5 µM). All data was processed in TraceDrawer (Reichert, 1.8.1) and double-referenced to buffer and a control channel that did not contain the immobilized binding partner. The association and dissociation phases of the HPF1 titration onto PARP1/DNA SSB was fit with a 1:1 binding model to obtain *k*_a_, *k*_d_, and *K*_D_ (Fig. 3c), and the equilibrium binding plateaus were used to obtain a *K*_D_ using a 1:1 binding model (Fig. 3b inset). Three independent experiments were performed and yielded the reported averages and standard deviations.

### Western blot assay

PARP1 WT (1 μM) was incubated with DNA (1 μM; dumbbell with a central nick) without or with HPF1 (0.1 μM) and histone octamer where indicated (1 μM) at RT for 10 min as described^46^. Reactions were started by adding NAD^+^ (1 mM) for various time points, stopped with PARP inhibitor talazoparib (500 μM) then treated with PARG (1 μM) for one hour at RT. SDS-PAGE (7.5%, 12% or gradient 4–20% from Bio-Rad) were run and the gels were transferred to a nitrocellulose membrane and the membrane was then blocked with 1% milk. The membrane was incubated with a mono-ADP-ribose binding reagent (MABE 1076, Millipore Sigma, 1:2500) or in the case of PARP3 with a pan ADP-ribose binding reagent (MABE 1016, Millipore Sigma, 1:1500) and next treated with a secondary antibody (donkey anti-rabbit conjugated to HRP, Santa Cruz, sc2313, 1:7000). The signal was revealed using ECL (Bio-Rad). Image J (1.53a) was used to quantify band intensities. In order to determine rates of initiation, a PARP1 standard reaction containing PARP1 (1 μM), DNA (1 μM), HPF1 (0.1 μM) and NAD^+^ (1 mM) was performed for 10 min at which point the initiation reaction is fully completed. The reaction was stopped and treated with PARG as described above. A series of dilutions of the standard reaction was performed to determine the linear range of detection. These dilutions were then included in each experiment to allow intensity units to be converted to pmol of ADP-ribose, based on the estimation of 3 dominant sites of Ser modification per PARP1 molecule in the presence of HPF1 (Ser 499, Ser 507, Ser 519)^35,36^. PARP1 time-course reactions with or without HPF1 were also diluted (150-fold or 20-fold, respectively) to ensure that the signal was in the linear range of the assay. A rate of initiation in 1/s was then calculated based on the standard curve and the amount of PARP1 in the reactions (12 pmol).

### Hydrogen/deuterium exchange mass spectrometry (HXMS)

Prior to deuterium on-exchange, 2.6  μM of PARP1 was incubated for 30 min with 5 μM of SSB DNA (5’ GCT GGC TTC GTA AGA AGC CAG CTC GCG GTC AGC TTG CTG ACC GCG)^9^. For the PARP1/DNA/HPF1 complex, 2.6 μM HPF1 was added to the mixture and incubated for another 30 min. Deuterium on-exchange was performed at room temperature by adding 5 μL of mixture to 15 μL of deuterium on-exchange buffer (10 mM HEPES, pD 7.0, 150 mM NaCl, in D_2_O, pD = pH + 0.4138) to yield a final D_2_O concentration of 75%. At specified time points, 20 μL aliquots were added into 30 μL of ice-cold quench buffer (1.66 M guanidine hydrochloride, 10% glycerol, and 0.8% formic acid to make a final pH of 2.4–2.5) and immediately frozen in liquid nitrogen until further use. Deuterium on-exchange was carried out in triplicate (*n* = 3) for all the time points (10 s, 100 s, 1000 s, 10,000 s, 100,000 s). The Non-deuterated (ND) samples of PARP1 were prepared in 10 mM HEPES, pH 7.0, 150 mM NaCl buffer and each 20 μL aliquot was quenched into 30 μL of quench buffer. To mimic the on-exchange experiment, the fully deuterated (FD) samples were prepared in 75% deuterium, but denatured under acidic conditions (0.5% formic acid). The samples were incubated for 48 h to ensure every amide proton along the entire polypeptide chain to undergo full exchange. Fifty microliters of samples were melted at 0 °C, rapidly injected into pepsin column and simultaneously pumped at initial flow rate of 50 μL min^−1^ for 2 min followed by 150 μL min^−1^ for another 2 min. Pepsin (Sigma) was coupled to POROS 20 AL support (Applied Biosystems) and the immobilized pepsin was packed into a 64 μL column (2 mm × 2 cm, Upchurch). The pepsin-digested peptides were trapped onto a TARGA C8 5 μm Piccolo HPLC column (1.0 × 5.0 mm, Higgins Analytical) and eluted through an analytical C18 HPLC column (0.3 × 75 mm, Agilent) with a 12–100% buffer B gradient at 6 μL/min (Buffer A: 0. 1% formic acid; Buffer B: 0. 1% formic acid, 99.9% acetonitrile). The effluent was electrosprayed into the Exactive Plus EMR-Orbitrap (Thermo Fisher Scientific). MS data acquisition over the mass range 200−2000 m/z were acquired on the Exactive Plus EMR-Orbitrap (Thermo Fisher Scientific) at 60,000 resolution. The effluent was electrosprayed with ion spray voltage of 3.5 kV and capillary temperature operated at 250 °C.

### PARP1 peptide identification

For peptide identification the ND samples were injected into LTQ orbitrap XL, (Thermo Fisher Scientific) for tandem mass spectroscopy (MS/MS). Scan range for MS/MS data was 200–2000 m/z at 15,000 resolution, where the ions were fragmented by CID with normalized collision energy 35. The potential PARP1 peptides were identified through SEQUEST (Bioworks v3.3.1) with a peptide tolerance of 8 ppm and a fragment tolerance of 0.1 AMU against an extensive decoy sequence database (custom database) containing the sequence of PARP1, pepsin and other common contaminants identified in prior HXMS studies. Since pepsin was employed as the protease, we specified it in our search as a non-specific digestion enzyme. After the MS/MS was performed for the first ND, a MATLAB-based program (9.2.0.556344), ExMS2 (version 2017-07-19)^47^ was used with *P*_pep_ score of 0.1 to identify the peptides and to generate an exclusion list. While performing MS/MS on the second ND the instrument employed this exclusion list to collect the MS2 scan of the less intense peptides that were not identified in the previous ND sample. At least four such exclusion lists were generated to increase the number of unique peptides and sequence coverage of the protein. Finally, pool of peptides based on SEQUEST output files were prepared by EXMS2.

### HXMS analysis and plotting

HDExaminer software (v 2.5.0) was used, which now uses the peptide pool information generated through ExMS2 to identify the deuterated peptides for every sample in the full time-course HXMS experiment. Each individual deuterated peptide is corrected for loss of deuterium label (back-exchange after quench) during HXMS data collection by normalizing to the maximal deuteration level of that peptide in the fully deuterated samples. For each peptide, we have compared the extent of deuteration as measured in the FD sample to the theoretical maximal deuteration (i.e., if no back-exchange occurs). The median extent of back-exchange in our experiments were in the 25-26% range Supplementary Fig. 8B). The data analysis statistics for all the protein states are in Supplementary Data 1 according to the suggestions of Masson et al.^48^. The quality of each peptide was further assessed by manually checking mass spectra. The mass spectrometry proteomics data have been deposited to the ProteomeXchange Consortium via the PRIDE^49^ partner repository with the dataset identifier PXD026580.

The difference plot for the deuteration levels between any two samples was obtained through an in-house script written in MATLAB. The script compares the deuteration levels between two samples (e.g., PARP1/DNA complex and PARP1/DNA/HPF1 complex) and plots the percent difference of each peptide, by subtracting the percent deuteration of PARP1/DNA/HPF1 complex from PARP-1/DNA complex and plotting according to the color legend in stepwise increments (as in Fig. 6a). The plot of representative peptide data is shown as the mean of three independent measurements + /- SD. Statistical analysis included a *t*-test with a *P* < 0.05 (as in Fig. 6b, c).

### Fluorescence polarization assay

The fluorescence polarization assay was performed as previously described using 5 nM of a dumbbell probe containing a central nick and an internal FAM group^34^ (5’GCT GAG C FAM-TT CTG GTG AAG CTC AGC TCG CGG CAG CTG GTG CTG CCG CGA), with or without HPF1 at 20 μM and increasing concentrations of PARP1 WT or R865A mutant from 1.56 nM to 400 nM. The *K*_D_ was calculated by fitting a 1:1 binding model to the data. At least three independent experiments were performed and the average and standard deviations are shown.

### Negative-stain EM

A PELCO easiGlow (Ted Pella Inc.) was used to glow discharge carbon-coated copper grids (Electron Microscopy Sciences) prior to sample adsorption. Purified PARP1 was assembled with SSB DNA and EB-47 with or without HPF1 at 5 μM in 25 mM Hepes pH 8.0, 150 mM NaCl, 0.1 mM TCEP buffer. PARP1-DNA-EB-47 (5 μΜ) and PARP1-DNA-EB47-HPF1 (2.5 μM) were adsorbed and stained with 1.5% uranyl formate using the “rapid flush” technique^50^. Grids were imaged at 67,000x magnification with a FEI Tecnai 12 transmission electron microscope operated at 120 keV using a LaB6 filament and FEI Eagle 4k CCD (Electron Imaging Facility, Faculty of Dental Medicine, Université de Montréal). A total of 158 (complex lacking HPF1) and 165 (complex with HPF1) micrographs were collected using the SerialEM program^51^. The micrographs were processed with RELION 3.1^52^ without contrast transfer function correction. Reference-free particle picking generated 203,168 (complex without HPF1) and 167,267 (complex with HPF1) particle coordinates. Pick locations were extracted in 80 pixel boxes and downsampled to 3.3 Å/pixel, corresponding to 264 Å box lengths. Iterative 2D classifications using 180 Å circular masks obtained a final dataset of 48,250 (without HPF1) and 25,335 (with HPF1) particles. The combined models presented in Fig. 7 were obtained by aligning a PARP1 Zn1/Zn3/WGR-CAT/DNA structure (4DQY) to a PARP1 Zn1/Zn2/DNA NMR structure (2N8A) using the Zn1 domain. PARP2 CATΔHD/HPF1 (6TX3) was aligned to 4DQY using the ART.

### Reporting summary

Further information on research design is available in the Nature Research Reporting Summary linked to this article.

## Supplementary information

Supplementary Information

Description of Additional Supplementary Files

Supplementary Data 1

Reporting Summary

## Data Availability

Source data are provided with this paper. The HXMS data in this study have been deposited in the Pride database under accession code PXD026580. The following structures from the Protein Data Bank (www.rcsb.org) were used in this study: 4DQY [10.2210/pdb4DQY/pdb] (PARP1/DNA crystal structure), 2N8A [10.2210/pdb2N8A/pdb] (PARP1 Zn1, Zn2/DNA NMR structure), 6TX3 [10.2210/pdb6TX3/pdb] (HPF1/PARP2 CATΔHD crystal structure). Source data are provided with this paper.

## Source data

Source Data
