# Supplementary material for: HPF1 dynamically controls the PARP1/2 balance between initiating and elongating ADP-ribose modifications
Source: Nat Commun. 2021 Nov 18;12:6675. doi: 10.1038/s41467-021-27043-8 (PMC8602370; doi:10.1038/s41467-021-27043-8)
Supplement: Supplementary file 1 — Supplementary Information [file 41467_2021_27043_MOESM1_ESM.pdf]

SUPPLEMENTARY INFORMATION

HPF1 dynamically controls the PARP1/2 balance between initiating and elongating ADP-ribose modifications

Marie-France Langelier, Ramya Billur, Aleksandr Sverzhinsky, Ben E. Black and John M. Pascal

Supplementary Figures 1 to 9

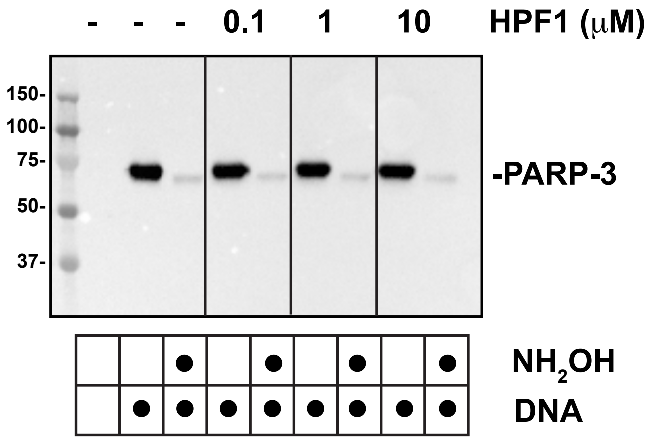

Supplementary Figure 1: HPF1 does not have an effect on PARP3 activity. PARP3 (1 μM) was incubated with a dumbbell DNA containing a central nick (1 μM) with a 5' Phosphate group (5'P) with or without HPF1 at various concentrations for 10 minutes at room temperature (RT). 500 μM NAD^+ was added for 15 minutes and reactions were quenched with 10 μM PARP inhibitor (veliparib). Where indicated, reactions were treated with 1M hydroxylamine (NH_2OH) for one hour. Reactions were run on SDS-PAGE and a western blot was performed using a pan ADP-ribose binding reagent. This experiment was confirmatory of published studies and thus performed one time. Source Data are provided as a Source Data file. Numbers on the left side of the blot represent molecular weight marker locations (values in kDa).

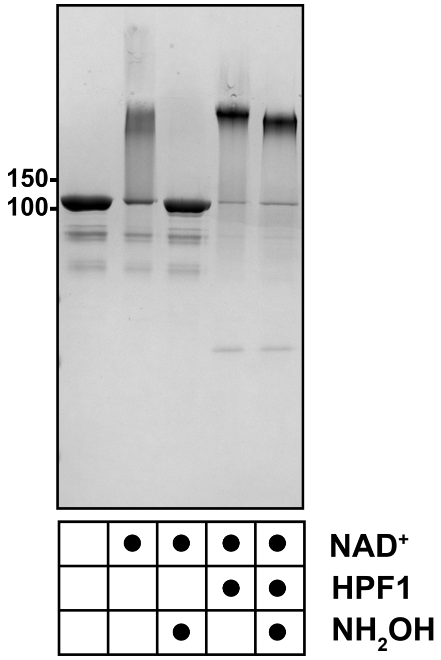

Supplementary Figure 2: HPF1 works at sub-stochiometric amount with the constitutively active PARP1 ΔHD in the absence of DNA. PARP1 ΔHD (1 μM) was incubated with or without HPF1 (0.1 μM) for 10 minutes at room temperature (RT). 500 μM NAD^+ was added for 5 minutes and reactions were quenched with 500 μM PARP inhibitor (talazoparib). Where indicated, reactions were treated with 1M hydroxylamine (NH_2OH) for one hour. Reactions were run on an SDS-PAGE and stained with Imperial Stain. This experiment was performed two times. Numbers on the left side of the gel represent molecular weight marker locations (values in kDa). Source Data are provided as a Source Data file.

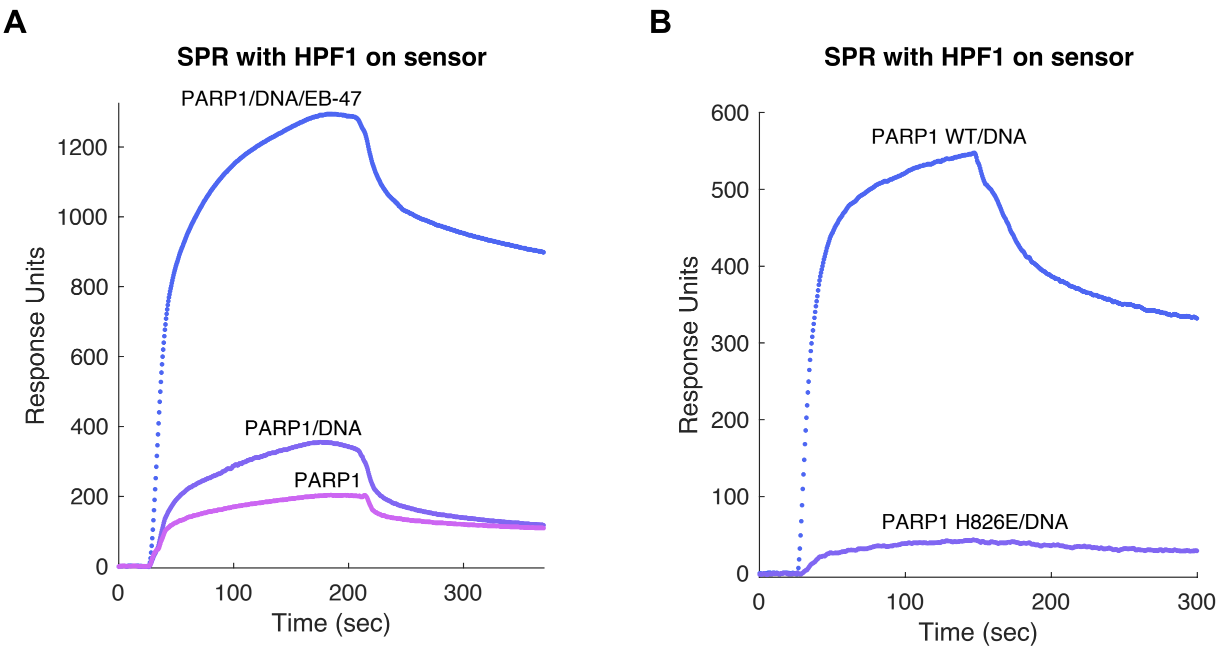

Supplementary Figure 3: HPF1 binds better to a PARP1/DNA complex in the presence of EB-47. A) HPF1 was immobilized on a biosensor chip by amine coupling. PARP1 (800 nM) was flowed over the HPF1-coated biosensor in the absence or presence of DNA (800 nM) and EB-47 (5 μM). B) In a separate experiment, PARP1 WT or H826E mutant (1 μM) in the presence of DNA (1 μM) were flowed over an HPF1 chip with EB-47 (5 μM) in the system buffer.

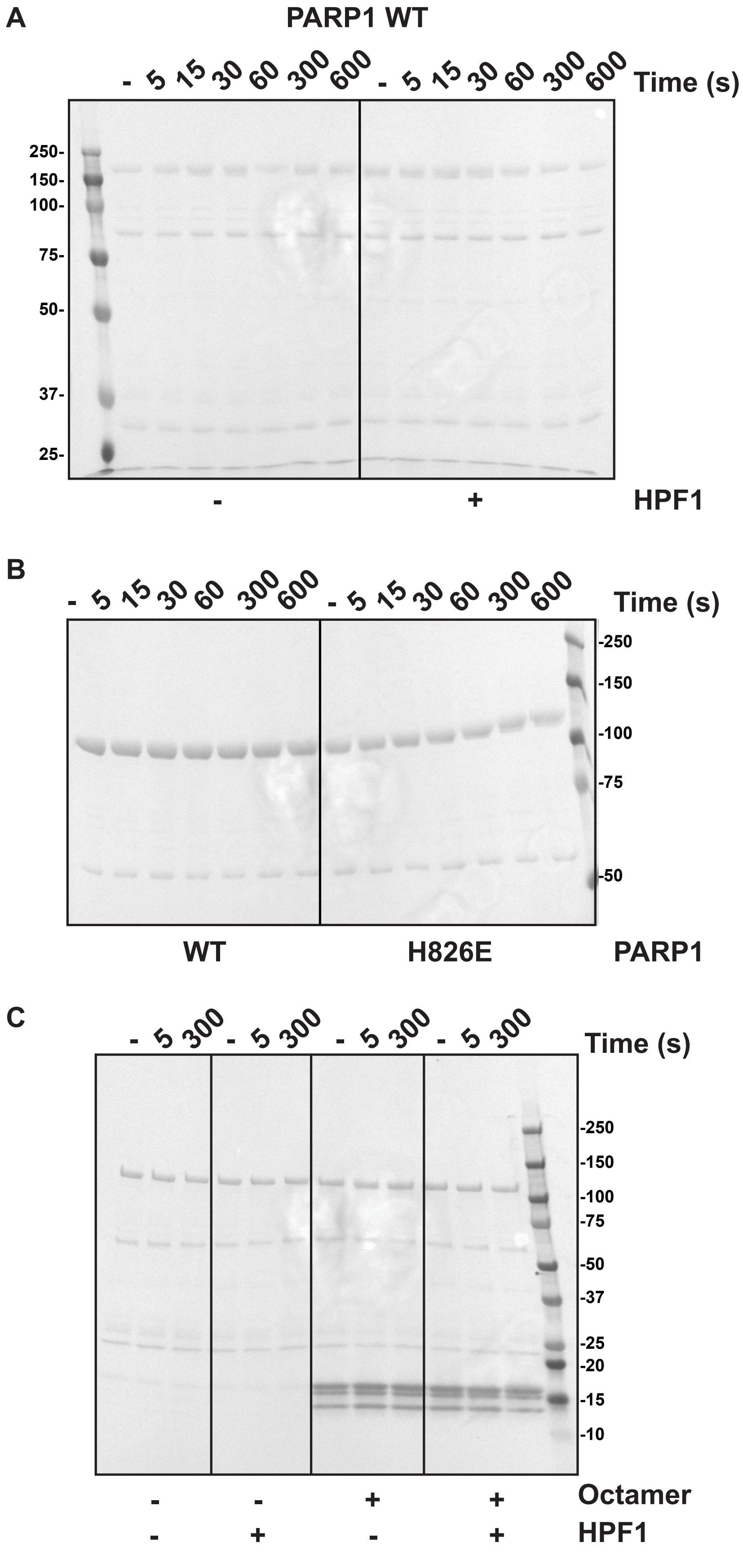
A
PARP1 WT
- 5 15 30 60 300 600 - 5 15 30 60 300 600 Time (s)
250-
150-
100-
75-
50-
37-
25-
- + HPF1
B
- 5 15 30 60 300 600 - 5 15 30 60 300 600 Time (s)
-250
-150
-100
-75
-50
WT H826E PARP1
C
- 5 300 - 5 300 - 5 300 - 5 300 Time (s)
-250
-150
-100
-75
-50
-37
-25
-20
-15
-10
- - + + Octamer
- + - + HPF1

Supplementary Figure 4: Western Blots loading controls. Ponceau-stained membranes corresponding to Western Blots shown in Figure 5 A, B and Supp Fig. 5C. These experiments were performed three times. Numbers on the left or right side of the membranes represent molecular weight marker locations (values in kDa). Source Data are provided as a Source Data file.

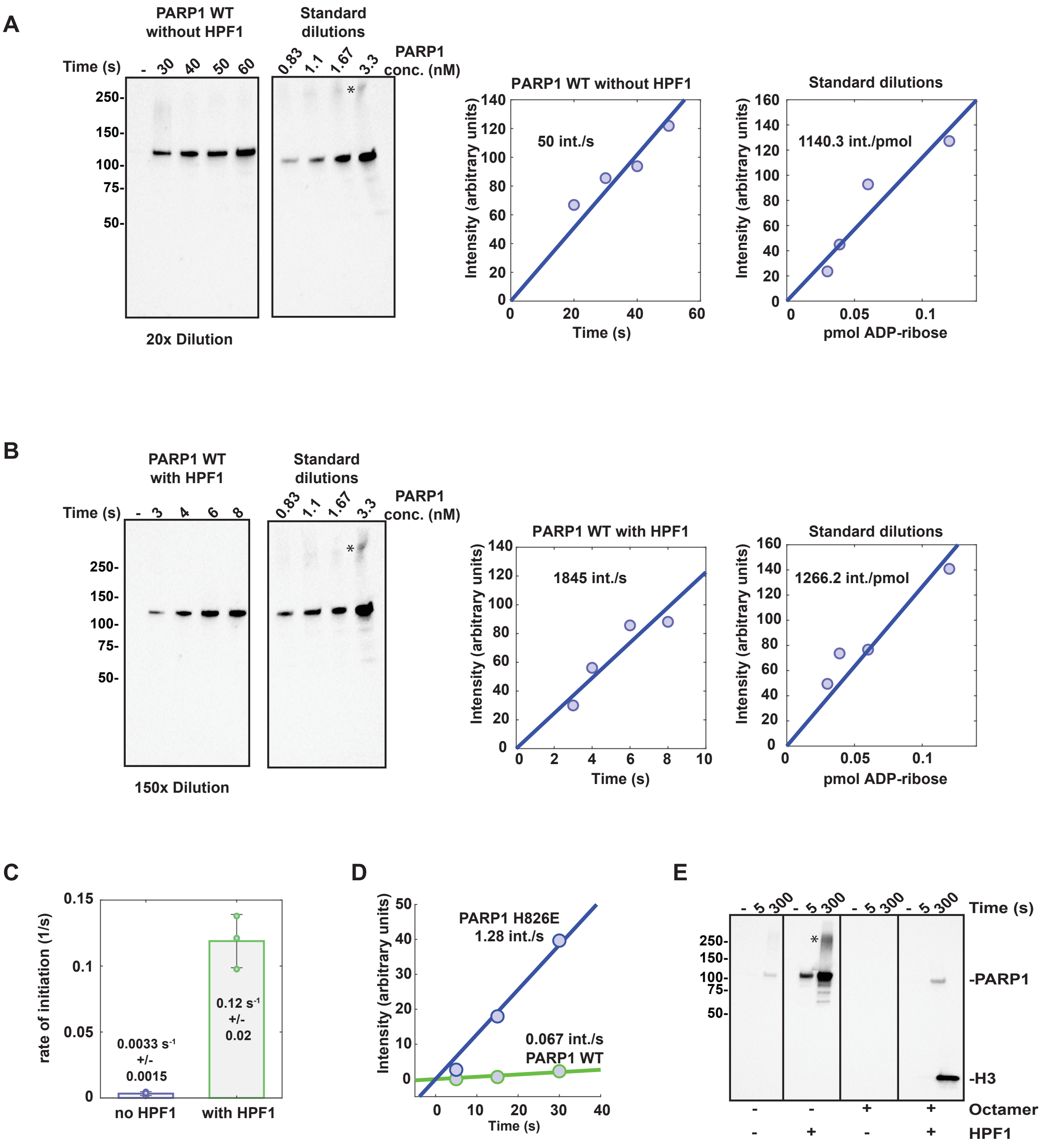

A
PARP1 WT without HPF1
Standard dilutions
Time (s)
- 30 40 50 60
0.83 1.1 1.67 3.3
PARP1 conc. (nM)
250-
150-
100-
75-
50-
*
20x Dilution
PARP1 WT without HPF1
50 int./s
Intensity (arbitrary units)
Time (s)
Standard dilutions
1140.3 int./pmol
pmol ADP-ribose
B
PARP1 WT with HPF1
Standard dilutions
Time (s)
- 3 4 6 8
0.83 1.1 1.67 3.3
PARP1 conc. (nM)
250-
150-
100-
75-
50-
*
150x Dilution
PARP1 WT with HPF1
1845 int./s
Intensity (arbitrary units)
Time (s)
Standard dilutions
1266.2 int./pmol
pmol ADP-ribose
C
rate of initiation (1/s)
0.0033 s-1 +/- 0.0015
0.12 s-1 +/- 0.02
no HPF1
with HPF1
D
Intensity (arbitrary units)
PARP1 H826E
1.28 int./s
0.067 int./s
PARP1 WT
Time (s)
E
- 5 300 - 5 300 - 5 300 - 5 300
Time (s)
250-
150-
100-
75-
50-
*
-PARP1
-H3
Octamer
HPF1

Supplementary Figure 5: HPF1 stimulates initiation by PARP1. A) Left panel (PARP1 WT without HPF1): PARP1 (1 μM) was incubated without HPF1 for 10 minutes at RT in the presence of DNA (1 μM). 1 mM NAD^+ was added for various time points as indicated and reactions were quenched with 500 μM Talazoparib. PARG (1 μM) was added and incubated for 1 hour at RT. Reactions were diluted 20-fold to stay in the linear range of the assay, and then resolved on 4 to 20% gradient SDS-PAGE gels followed by a western blot using a mono ADP-ribose binding reagent. Right panel (Standard dilutions): A reaction containing PARP1 (1 μM), HPF1 (0.1 μM), DNA (1 μM) and 1 mM NAD^+ was performed until saturation (10 minutes) and diluted as indicated to provide a standard curve. These dilutions were used as a standard to calculate the amount of pmol of ADP-ribose per intensity unit. We estimated that in the presence of HPF1, there are 3 main sites of ADP-ribose modification on PARP1 (Ser 499, Ser 507 and Ser 519). The bands corresponding to mono ADP-ribosylated PARP1 were quantified using ImageJ and plotted. B) Same as in panel A, but in the presence of HPF1 (0.1 μM) and the reactions were diluted 150-fold instead of 20-fold. C) The average initiation rates (s^{-1}) of 3 independent initiation experiments as described in A) and B) were calculated by dividing the rate in pmol of ADP-ribose per s by the amount of PARP1 in the reaction (12 pmol). The standard deviation and the individual points of each experiments are shown. D) Early time-points in the linear region from the experiment shown in the main Figure 5B were used to estimate a fold rate change between the PARP1 WT versus H826E reactions. E) Reactions were performed as in Figure 5A but in the presence of histone octamer, where indicated. In all panels, the additional bands (denoted *) likely correspond to very large, undigested PAR molecules that are usually not observed when using 12% gels, where they would not enter the resolving gel. All experiments in this figure were performed three times. Numbers on the left side of the membranes represent molecular weight marker locations (values in kDa). Source Data are provided as a Source Data file.

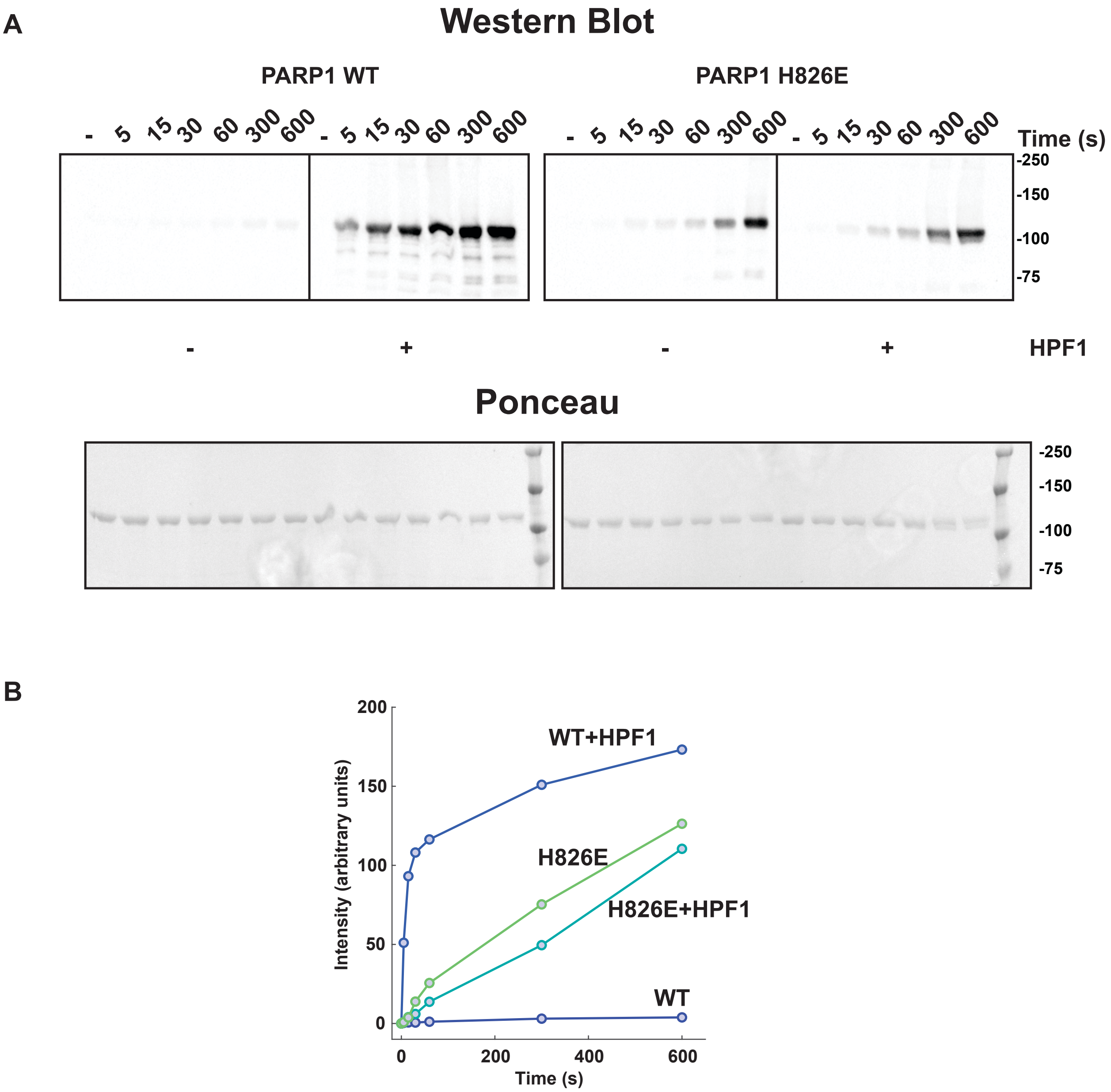

Supplementary Figure 6: HPF1 does not stimulate PARP1 mutant H826E. A) PARP1 WT or H826E mutant (1 μM) was incubated without or with HPF1 (0.1 μM) for 10 minutes at RT in the presence of DNA (1 μM). 1 mM NAD^+ was added for various time points and reactions were quenched with 500 μM Talazoparib. PARG (1 μM) was added and incubated for 1 hour at RT. Reactions were run on SDS-PAGE. The gels were cut so they could be transferred on the same membrane so that the signals could be compared and a western blot was performed using a mono ADP-ribose binding reagent. This experiment was performed three times. B) The bands corresponding to mono ADP-ribosylated PARP1 were quantified using ImageJ and the intensities were plotted over time. Numbers on the right side of the membranes represent molecular weight marker locations (values in kDa). Source Data are provided as a Source Data file.

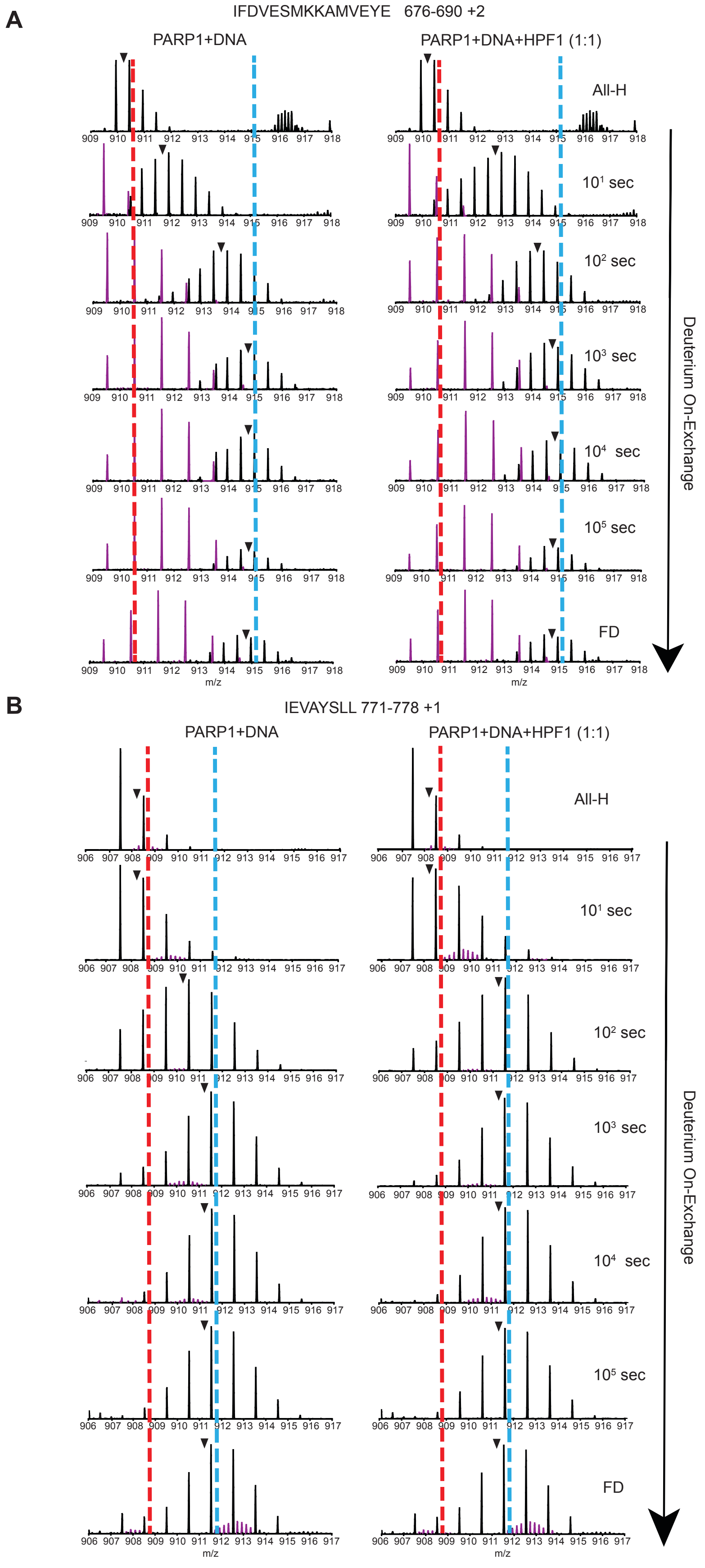
A
IFDVESMKKAMVEYE 676-690 +2
PARP1+DNA
PARP1+DNA+HPF1 (1:1)
All-H
10¹ sec
10² sec
10³ sec
10⁴ sec
10⁵ sec
FD
Deuterium On-Exchange
m/z
B
IEVAYSLL 771-778 +1
PARP1+DNA
PARP1+DNA+HPF1 (1:1)
All-H
10¹ sec
10² sec
10³ sec
10⁴ sec
10⁵ sec
FD
Deuterium On-Exchange
m/z

Supplementary Figure 7: Raw MS data of representative peptide from αB and αF helices of HD. Spectra for PARP1/DNA complex and PARP1/DNA/HPF1 complex for the full-time course HXMS experiment is shown. All- H represents the non-deuterated sample. FD represents the fully-deuterated sample. Black isotopic envelopes represent the αB peptide (A) and αF helices (B) of HD, whereas purple isotopic envelopes in the same m/z region represents the co-eluting peptides with a different charge state. Black triangles indicate the centroid value. Red and blue dotted lines visualize the differences in m/z for the representative peptides of αB peptide and αF helices of HD.

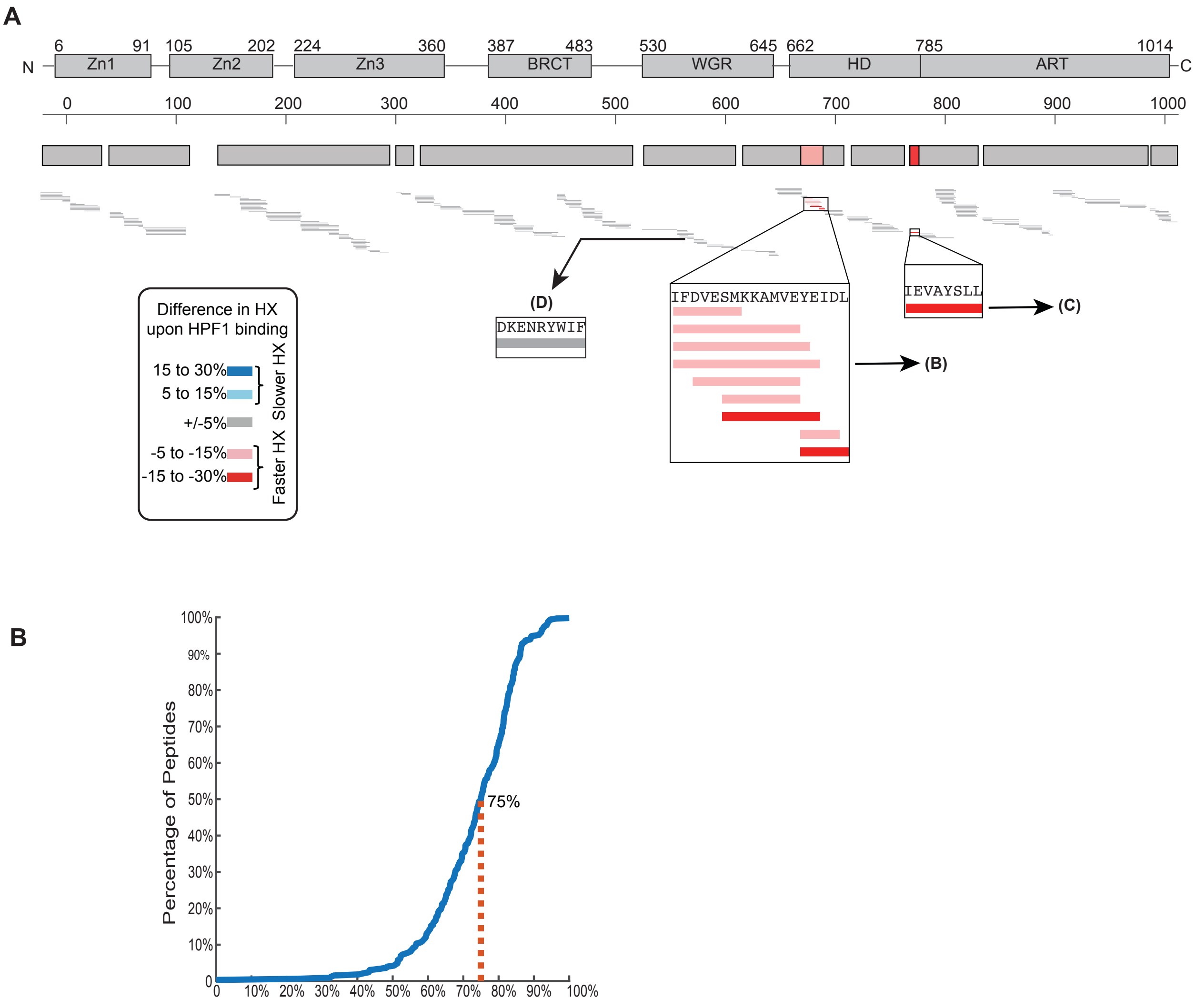

Supplementary Figure 8: HXMS analysis of HPF1 interaction with PARP1 bound to a DNA SSB at 100 s. A) The difference plot was obtained by subtracting the percent deuteration of PARP1/DNA/HPF1 complex from PARP-1/DNA complex at 100 s. Each horizontal bar represents a peptide. The difference plot indicates that most of the peptides (grey) in PARP1/DNA/HPF1 complex have similar HX rates when compared to PARP1/DNA complex. However, peptides in αB and αF helices of HD showed faster exchange (red). The white regions in the difference plot represent gaps in the peptide coverage. B) Distribution curve of a representative FD sample. The deuteration was ~75% for the FD sample and the median back-exchange after the quenching was ~25%, which is within the acceptable range. The extent of deuteration of each peptide is calculated as [(FD centroid – ND centroid) * cs] / [maxD * fracDeut], cs is the charge state, maxD is the number of residues minus first two residues (back-exchange within experimental timescale) and minus number of prolines due to no backbone amide hydrogen, and fracDeut = 0.75 (fraction of deuterium during on-exchange).

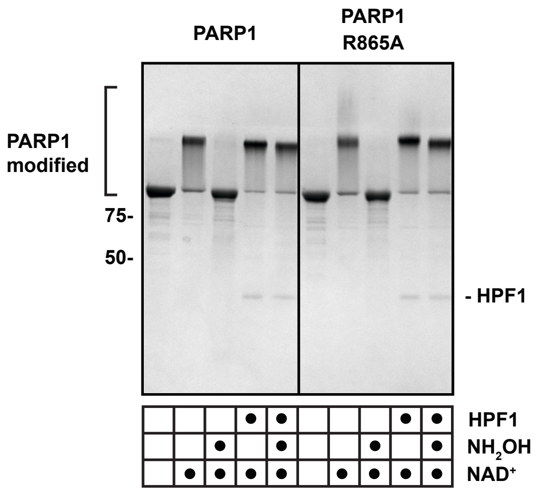

Supplementary Figure 9: PARP1 R865A is able to automodify on Ser in the presence of HPF1. PARP1 WT or mutant R865A (1 μM) was incubated with or without HPF1 (0.1 μM) for 10 minutes at room temperature (RT). 500 μM NAD^+ was added for 5 minutes and reactions were quenched with 500 μM PARP inhibitor (talazoparib). Where indicated, reactions were treated with 1M hydroxylamine (NH_2OH) for one hour. Reactions were run on an SDS-PAGE and stained with Imperial Stain. Numbers on the left side of the gel represent molecular weight marker locations (values in kDa). This experiment was performed twice. Source Data are provided as a Source Data file.
