# Supplementary material for: HPF1 dynamically controls the PARP1/2 balance between initiating and elongating ADP-ribose modifications
Source: Nat Commun. 2021 Nov 18;12:6675. doi: 10.1038/s41467-021-27043-8 (PMC8602370; doi:10.1038/s41467-021-27043-8)
Supplement: Supplementary file 2 — Description of Additional Supplementary Files [file 41467_2021_27043_MOESM2_ESM.docx]

**Description of Additional Supplementary Files**

**File Name:** Supplementary Data 1

**Description:** HXMS Data Summary and Results.
