# Supplementary material for: HPF1 dynamically controls the PARP1/2 balance between initiating and elongating ADP-ribose modifications
Source: Nat Commun. 2021 Nov 18;12:6675. doi: 10.1038/s41467-021-27043-8 (PMC8602370; doi:10.1038/s41467-021-27043-8)
Supplement: Supplementary file 4 — Reporting Summary [file 41467_2021_27043_MOESM4_ESM.pdf]

nature research

Corresponding author(s): John Pascal

Last updated by author(s): Oct 22, 2021

Reporting Summary

Nature Research wishes to improve the reproducibility of the work that we publish. This form provides structure for consistency and transparency in reporting. For further information on Nature Research policies, see our Editorial Policies and the Editorial Policy Checklist.

Statistics

For all statistical analyses, confirm that the following items are present in the figure legend, table legend, main text, or Methods section.

n/a	Confirmed	
☐	☒	The exact sample size (*n*) for each experimental group/condition, given as a discrete number and unit of measurement
☐	☒	A statement on whether measurements were taken from distinct samples or whether the same sample was measured repeatedly
☐	☒	The statistical test(s) used AND whether they are one- or two-sided<br>*Only common tests should be described solely by name; describe more complex techniques in the Methods section.*
☒	☐	A description of all covariates tested
☒	☐	A description of any assumptions or corrections, such as tests of normality and adjustment for multiple comparisons
☐	☒	A full description of the statistical parameters including central tendency (e.g. means) or other basic estimates (e.g. regression coefficient) AND variation (e.g. standard deviation) or associated estimates of uncertainty (e.g. confidence intervals)
☐	☒	For null hypothesis testing, the test statistic (e.g. *F*, *t*, *r*) with confidence intervals, effect sizes, degrees of freedom and *P* value noted<br>*Give P values as exact values whenever suitable.*
☒	☐	For Bayesian analysis, information on the choice of priors and Markov chain Monte Carlo settings
☒	☐	For hierarchical and complex designs, identification of the appropriate level for tests and full reporting of outcomes
☒	☐	Estimates of effect sizes (e.g. Cohen's *d*, Pearson's *r*), indicating how they were calculated

Our web collection on statistics for biologists contains articles on many of the points above.

Software and code

Policy information about availability of computer code

Data collection	Reichert4SPR (2.0.2.14) was used to collect SPR data.
Data analysis	Trace Drawer (Reichert) software (1.8.1) was used to analyze the SPR data. For HXMS, SEQUEST (Bioworks v3.3.1 was used to identify the likely sequence of parent peptides. A MATLAB based program, ExMS2 (version date: 2017-07-19) was used to identify the parental non-deuterated peptides through peptide envelope centroid values and chromatographic elution time ranges. HDExaminer software (v 2.5.0) was used to identify the deuterated peptides. Image J (1.53a) was used to quantify Western Blots bands.

For manuscripts utilizing custom algorithms or software that are central to the research but not yet described in published literature, software must be made available to editors and reviewers. We strongly encourage code deposition in a community repository (e.g. GitHub). See the Nature Research guidelines for submitting code & software for further information.

Data

Policy information about availability of data

All manuscripts must include a data availability statement. This statement should provide the following information, where applicable:

- Accession codes, unique identifiers, or web links for publicly available datasets
- A list of figures that have associated raw data
- A description of any restrictions on data availability

Source data are provided with this paper. The HXMS data in this study have been deposited in the Pride database under accession code PXD026580 (https://www.ebi.ac.uk/pride/archive/projects/PXD026580). The following structures from the Protein Data Bank (www.rcsb.org) were used in this study: 4DQY, 2N8A, 6TX3.

Field-specific reporting

Please select the one below that is the best fit for your research. If you are not sure, read the appropriate sections before making your selection.

- [x] Life sciences
- [ ] Behavioural & social sciences
- [ ] Ecological, evolutionary & environmental sciences

For a reference copy of the document with all sections, see nature.com/documents/nr-reporting-summary-flat.pdf

Life sciences study design

All studies must disclose on these points even when the disclosure is negative.

Sample size	No statistical methods were used to determine sample size since no inferences were made from small sample numbers to larger populations. All experiments were repeated two or three times independently to ensure reproducibility, which is the standard practice in the field.
Data exclusions	No data were excluded.
Replication	All experiments were replicated as independent experiments two or three times and yielded reproducible results.
Randomization	No randomization was required due to the nature of the study which does not involve human subjects, other organisms or compounds to be tested but rather involves biochemical assays with purified components that could not be randomized.
Blinding	No blinding was implemented. Samples and controls were treated and analyzed following the exact same procedure. The risk of bias is considered limited due to the nature of the experiments performed, and there was no preconceived outcome of the study.

Reporting for specific materials, systems and methods

We require information from authors about some types of materials, experimental systems and methods used in many studies. Here, indicate whether each material, system or method listed is relevant to your study. If you are not sure if a list item applies to your research, read the appropriate section before selecting a response.

Materials & experimental systems

n/a	Involved in the study	
☐	☒	Antibodies
☒	☐	Eukaryotic cell lines
☒	☐	Palaeontology and archaeology
☒	☐	Animals and other organisms
☒	☐	Human research participants
☒	☐	Clinical data
☒	☐	Dual use research of concern

Methods

n/a	Involved in the study	
☒	☐	ChIP-seq
☒	☐	Flow cytometry
☒	☐	MRI-based neuroimaging

Antibodies

Antibodies used	Mono ADP-ribose binding reagent (MABE 1076, Millipore Sigma). Pan ADP-ribose binding reagent (MABE 1016, Millipore Sigma). Secondary antibody (donkey anti-rabbit conjugated to HRP, Santa Cruz, sc2313)
Validation	All the antibodies were validated by the Manufacturers by Western Blotting. See images for the each antibody on the Manufacturers website using the links provided. MABE 1076: https://www.emdmillipore.com/CA/en/product/Anti-mono-ADP-ribose-binding-reagent,MM_NF-MABE1076#overview<br>MABE 1016: https://www.emdmillipore.com/CA/en/product/Anti-pan-ADP-ribose-binding-reagent,MM_NF-MABE1016<br>sc2313: https://www.scbt.com/p/donkey-anti-rabbit-igg-hrp
